# Supplementary material for: Novel gene therapy for drug-resistant melanoma: Synergistic combination of PTEN plasmid and BRD4 PROTAC-loaded lipid nanocarriers
Source: Mol Ther Nucleic Acids. 2024 Jul 31;35(3):102292. doi: 10.1016/j.omtn.2024.102292 (PMC11374965; doi:10.1016/j.omtn.2024.102292)
Supplement: Document S1. Figures S1–S6 and Table S1 [file mmc1.pdf]

OMTN, Volume *35*

Supplemental information

Novel gene therapy for drug-resistant melanoma: Synergistic combination of PTEN plasmid and BRD4 PROTAC-loaded lipid nanocarriers

Aishwarya Saraswat, Hari Priya Vemana, Vikas Dukhande, and Ketan Patel

Methods

HPLC method development for ARV-825

The chromatographic detection of ARV was analyzed using Waters alliance system equipped with 2998 Photodiode Array (PDA) detector and InertSustain C18 column (150 mm × 4.6 mm, 5 µm) (GL Sciences, CA, USA). Acetonitrile and potassium dihydrogen phosphate buffer of pH 3.5 at a volume ratio of 60:40 was used as the mobile phase at a flow rate of 1 mL/min and the injection volume was 10 µL. The output signal was monitored and processed using Empower 3 software. The column temperature was kept at 25°C and ARV was detected at a wavelength of 247 nm. The optimized mobile phase ratio resulted in a sharp peak of ARV with a retention time of 6.7 ± 0.2 min as shown in Fig. S2.

Table S1. *IC_{50} values of ARV alone and in combination with PTEN transfected via Transit LT1 in BRAFi-resistant melanoma cell lines. (n = 6, Data are represented as mean ± SD).*

**Cell line**	**IC_{50} of ARV (μM)**		**Fold decrease in IC_{50} of ARV**
	**ARV alone**	**ARV+PTEN**	
A375V	0.08 ± 0.01	0.01 ± 0.005	~8.0
RPMI-7951	0.50 ± 0.11	0.014 ± 0.003	~15.0

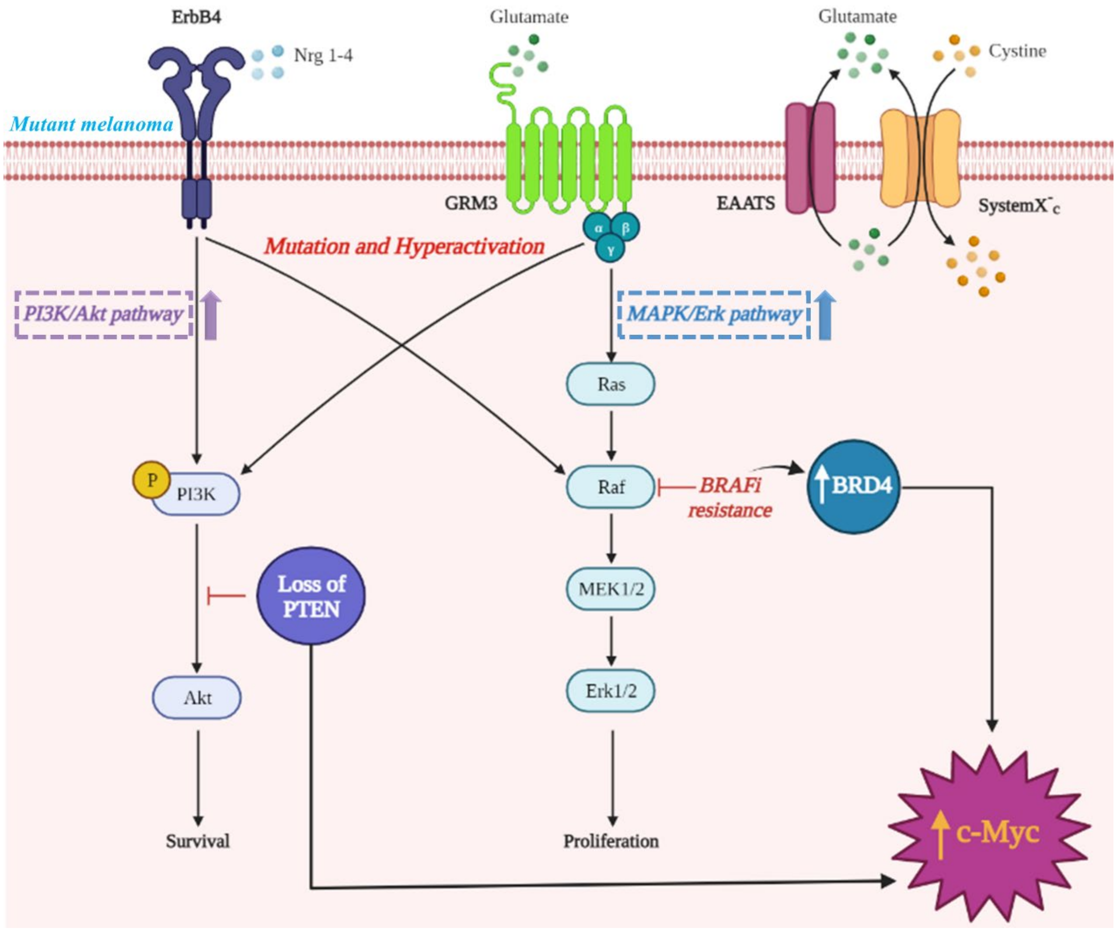

Fig. S1. Targeting of two key signaling pathways responsible for development of resistance against BRAFi in mutant melanoma. *Hyperactivation of major cellular signaling pathways including MAPK/ERK and PI3K/Akt pathways result in upregulation of BRD4 protein and loss of PTEN gene, respectively, to finally converge to c-Myc activation.*

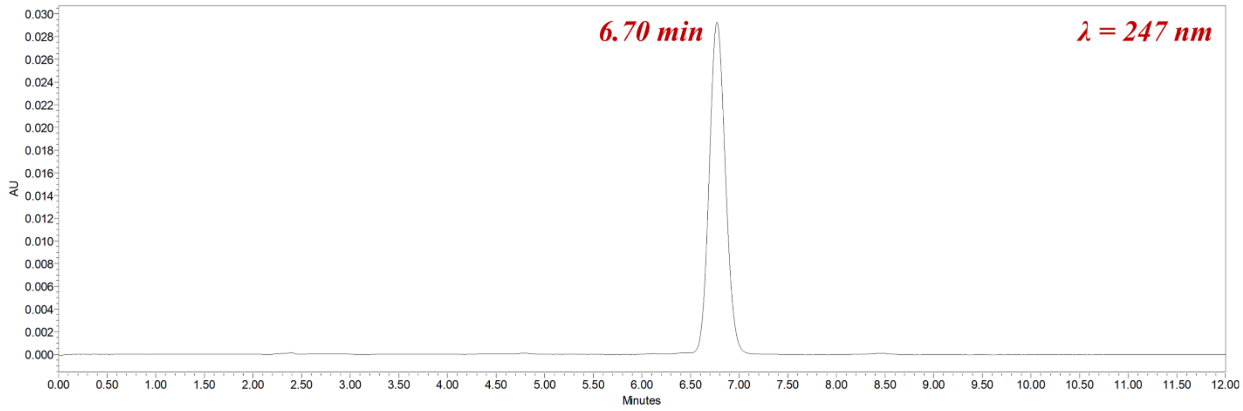

Fig. S2. Chromatogram of ARV corresponding to 10 ppm concentration as analyzed by HPLC. *ARV was found to be retained at 6.70 min to give a sharp peak when detected at a wavelength of 247 nm.*

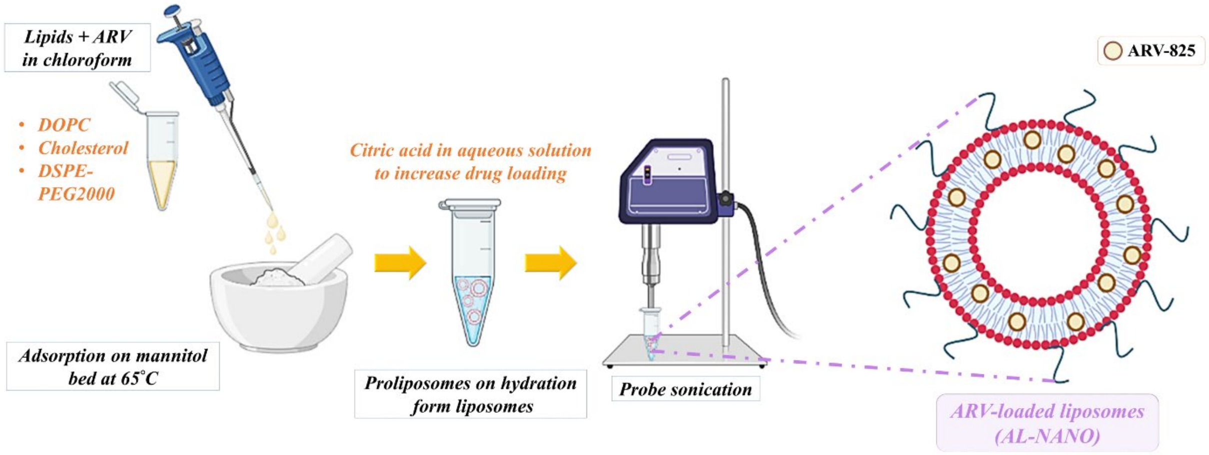

Fig. S3. Schematic representation for preparation of ARV-loaded liposomes (AL-NANO) by modified hydration method. *Lipids and ARV were dissolved in chloroform and adsorbed on mannitol bed. Following evaporation of organic solvent, resultant proliposomes were hydrated with citric acid solution and probe sonicated to form ARV-NANO that were used to perform in vitro assays.*

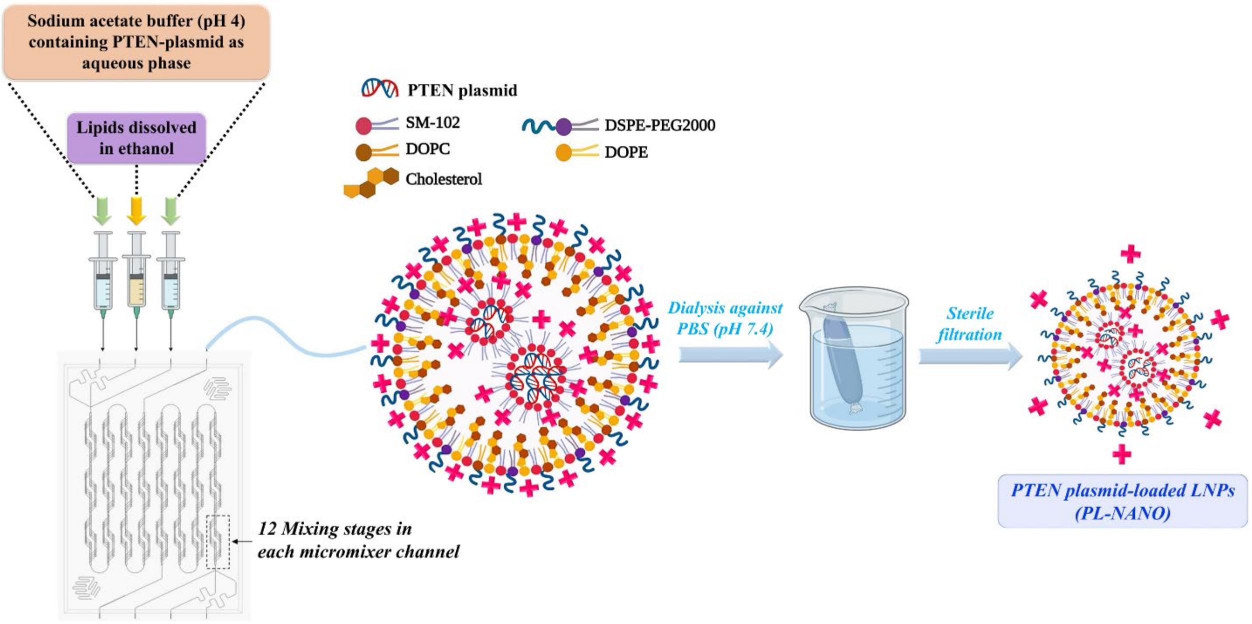

Fig. S4. Schematic representation for preparation of PTEN plasmid-loaded LNPs (PL-NANO) by microfluidic mixing approach. *Lipids dissolved in ethanol and PTEN-pDNA in buffer as aqueous solution are pumped into the two inlets of the microfluidic mixer using a syringe pump. Staggered herringbone structures induce chaotic mixing of both phases and correspondingly rapid increases in the polarity of the lipid solution to form PL-NANO. PL-NANO are further subjected to dialysis and sterile filtration for in vitro experiments.*

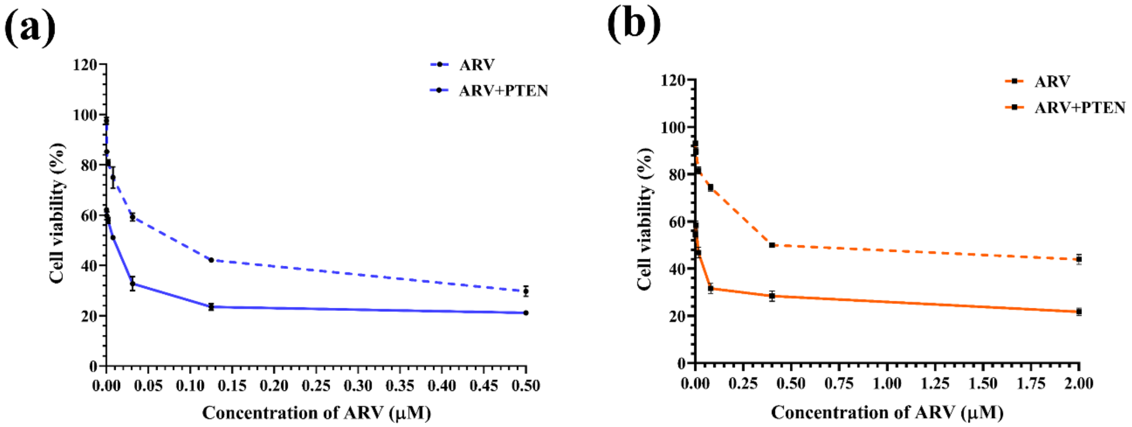

Fig. S5. Cytotoxicity curves of ARV and PTEN combination in VEM-resistant melanoma cell lines. *MTT cytotoxicity curves in* ***(a)*** *A375V (acquired) and* ***(b)*** *RPMI-7951 (intrinsic) resistant cell lines indicating strong synergism between ARV and PTEN in BRAFi-resistant melanoma. Control: Non-treated cells. (n = 6, Data are represented as mean ± SD).*

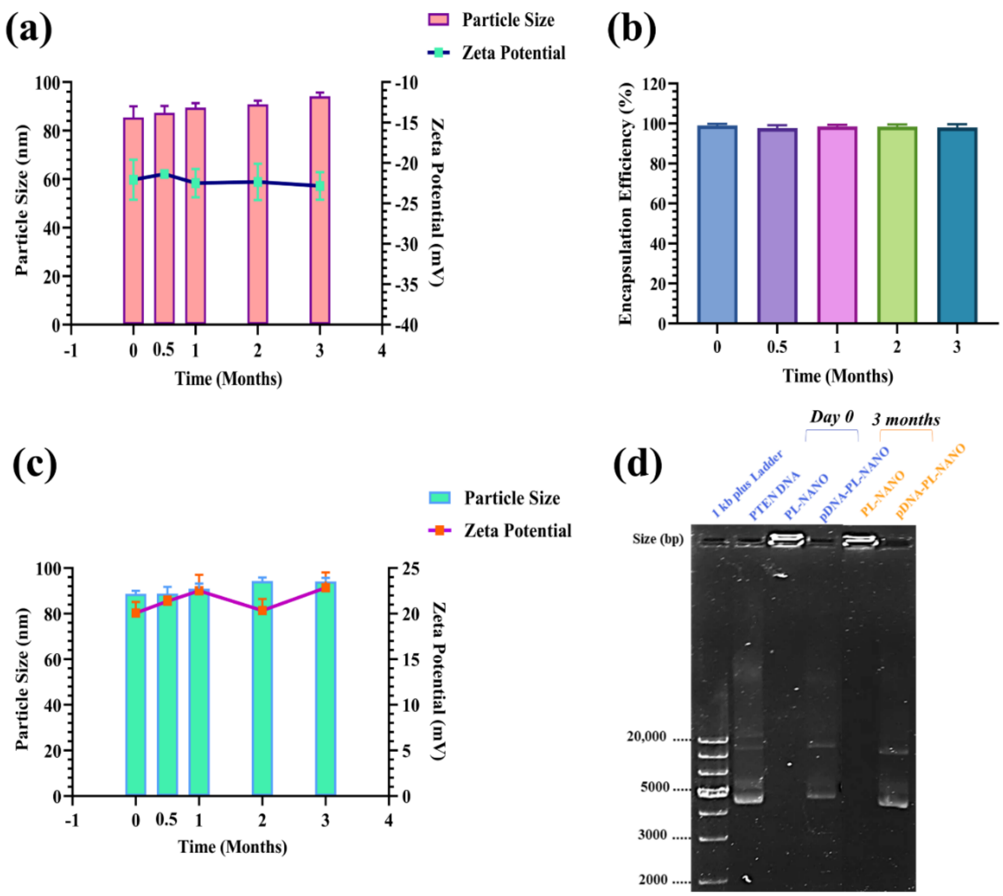

Fig. S6. Stability of AL-NANO and PL-NANO nanoformulations. (a) *Particle size and zeta potential of AL-NANO at different time points indicating no significant difference.* ***(b)*** *Entrapment efficiency of ARV in AL-NANO following 3 months at 4°C.* ***(c)*** *Particle size and zeta potential of PL-NANO depicting no substantial change at different time points.* ***(d)*** *Representative agarose gel electrophoresis image illustrating complete entrapment of PTEN plasmid in PL-NANO following 3 months at 4°C. (n = 3, Data are represented as mean ± SD).*
