# Supplementary material for: Novel gene therapy for drug-resistant melanoma: Synergistic combination of PTEN plasmid and BRD4 PROTAC-loaded lipid nanocarriers
Source: Mol Ther Nucleic Acids. 2024 Jul 31;35(3):102292. doi: 10.1016/j.omtn.2024.102292 (PMC11374965; doi:10.1016/j.omtn.2024.102292)
Supplement: Document S2. Article plus supplemental information [file mmc2.pdf]

Molecular Therapy
Nucleic Acids
Original Article

Novel gene therapy for drug-resistant melanoma: Synergistic combination of PTEN plasmid and BRD4 PROTAC-loaded lipid nanocarriers

Aishwarya Saraswat,[1] Hari Priya Vemana,[1] Vikas Dukhande,[1] and Ketan Patel[1]

[1]College of Pharmacy and Health Sciences, St. John's University, Queens, NY 11439, USA

Patients suffering from BRAF mutant melanoma have tumor recurrence within merely 7 months of treatment with a potent BRAF inhibitor (BRAFi) like vemurafenib. It has been proven that diverse molecular pathways driving BRAFi resistance converge to activation of c-Myc in melanoma. Therefore, we identified a novel combinatorial therapeutic strategy by targeting loss of phosphatase and tensin homolog deleted on chromosome 10 (PTEN) tumor suppressor gene and upregulated BRD4 oncoprotein as Myc-dependent vulnerabilities of drug-resistant melanoma. Being promising therapeutic targets, we decided to concomitantly deliver PTEN plasmid and BRD4 targeted PROteolysis-TArgeting Chimera (ARV) to drug the "undruggable" c-Myc in BRAFi-resistant melanoma. Since PTEN plasmid and ARV are distinct in their physicochemical properties, we fabricated PTEN-plasmid loaded lipid nanoparticles (PL-NANO) and ARV-825-loaded nanoliposomes (AL-NANO) to yield a mean particle size of less than 100 nm and greater than 99% encapsulation efficiency for each therapeutic payload. Combination of PL-NANO and AL-NANO displayed synergistic tumor growth inhibition and substantial apoptosis in *in vitro* two-dimensional and three-dimensional models. Importantly, simultaneous delivery of PL-NANO and AL-NANO achieved significant upregulation of PTEN expression levels and degradation of BRD4 protein to ultimately downregulate c-Myc levels in BRAFi-resistant melanoma cells. Altogether, lipid nanocarriers delivering this novel lethal cocktail stands as one-of-a-kind gene therapy to target undruggable c-Myc oncogene in BRAFi-resistant melanoma.

INTRODUCTION

Melanoma is a type of malignant tumor derived from melanocytes present in the skin and is one of the most lethal forms of cancer worldwide. According to the American Cancer Society, 97,610 new cases and 7,990 deaths are projected to occur due to melanoma in the United States in 2023.[1] The US Food and Drug Administration (FDA) has approved several therapies for the treatment and management of melanoma over the past years, including chemotherapy, targeted therapy, and immunotherapy. However, melanoma is usually refractory to conventional chemotherapeutic agents.[2] This led to the identification of novel targeted therapy and new forms of immunotherapy for the treatment of advanced melanoma. Given that BRAF mutations occur in more than 50% of melanoma cases, specific and potent BRAF inhibitors (BRAFis) like vemurafenib (VEM) evolved as targeted therapy for BRAFV600E mutant melanoma by curbing the mitogen-activated protein kinases/extracellular signal-regulated kinases (MAPK/ERK) pathway. However, it has been widely reported that most patients have tumor recurrence after 7–9 months of treatment with VEM. Targeted therapies become less effective after prolonged treatment and with subsequent stages of melanoma due to acquired resistance to BRAFi. This occurs as a result of mutations, re-activation of key pathways including MAPK/ERK and phosphatidylinositol 3-kinase/protein kinase B (PI3K/Akt), and changes in the cells' interactions with the tumor microenvironment in melanoma tumors. Interestingly, a variety of mutations and switching of key cellular signaling pathways control cell proliferation rate, differentiation, and invasion, allowing melanoma cells to robustly resist targeted therapies.[3] Given this, acquired and inherited resistance to VEM severely limits its long-term clinical effects.[4]

Extensive studies have recognized diverse pathways that drive the resistance against BRAFi in BRAF-mutant melanoma, denoting that durable control of resistance would be challenging. It has been previously identified that most of these pathways converge to activation of oncogenic c-MYC to confer both inherent and acquired resistance against targeted therapy like BRAFi in mutant melanoma. Recently, a study by Singleton et al.[5] proved that c-Myc is the convergent downstream effector of multiple major signaling pathways including MAPK/ERK, PI3K/Akt, Notch1, and Yes-associated protein, that is both necessary and sufficient for BRAFi resistance in melanoma patients. Expression of c-Myc is found to be suppressed after drug treatment, while it is rebounded after the development of resistance and during progression of melanoma. Therefore, we investigated novel therapeutic strategies for suppression of oncogenic c-Myc activity via different genetic or epigenetic approaches to target BRAFi resistance in melanoma. Consequently, two critical pathways and associated dysregulated proteins were recognized that converged to

Received 4 February 2024; accepted 29 July 2024;
https://doi.org/10.1016/j.omtn.2024.102292.

Correspondence: Ketan Patel, College of Pharmacy and Health Sciences, St. John's University, Queens, NY 11439, USA.
E-mail: patelk2@stjohns.edu

Published by Elsevier Inc. on behalf of The American Society of Gene and Cell Therapy.
This is an open access article under the CC BY-NC-ND license (http://creativecommons.org/licenses/by-nc-nd/4.0/).

activation of c-Myc oncogene in BRAFi-resistant melanoma, as shown in Figure S1. Hyperactivation of PI3K/Akt pathway results in loss of phosphatase and tensin homolog deleted on chromosome 10 (PTEN) tumor suppressor gene and phosphorylation of downregulation proteins for prolonged survival of melanoma. While hyperactivation of MAPK/ERK pathway BRAF mutation results in upregulation of Bromodomain and extraterminal domain proteins like BRD4 in both primary and metastatic melanoma tissues. Both these key pathways finally converge to upregulate the expression of c-Myc.

Researchers have reported significant upregulation in levels of epigenetic BRD4 protein in primary and metastatic melanoma tissues, making it an attractive therapeutic target. For this reason, we used the target BRD4 protein degradation strategy to downregulate c-Myc in BRAFi-resistant melanoma. PROteolysis TArgeting Chimeras (PROTACs) are target protein degraders that consist of two high-affinity binding ligands, a ligand that binds to the target protein of interest connected via a linker to another ligand for an E3 ubiquitin ligase. Formation of this ternary complex leads to polyubiquitination and consequent proteasomal degradation of target protein at nanomolar potency via PROTACs.[6–8] In this study, ARV-825 (ARV) was selected as specific BRD4-targeted PROTAC to achieve target degradation and downregulation of BRD4 protein following subsequent suppression of c-Myc levels in VEM-resistant melanoma.[9,10] ARV has been proven to exhibit potent anticancer activity as a single agent or in combination with other tumor targeting small molecules in VEM-resistant melanoma cell lines *in vitro*.[11,12] Therefore, we aimed to investigate the antitumor potential of ARV by further combining it with other therapeutic modality to synergistically target oncogenic c-Myc expression in drug-resistant melanoma. Melanoma progression and development of resistance against BRAFi is closely associated with the loss of tumor suppressor gene, PTEN.[13] Being the second most mutated or deleted tumor suppressor after p53, PTEN is a dual lipid and protein phosphatase. Hence, the loss of PTEN is majorly responsible for the dysregulation of PI3K/Akt and RAS/MAPK pathways in BRAF mutant melanoma.[14,15] Dual targeting of both these pathways would therefore converge to ultimately targeting the suppression of c-Myc levels in drug-resistant melanoma. Given this significance, we chose PTEN tumor suppressor gene to combine with ARV for improved therapeutic potency and cooperative suppression of oncogenic c-Myc levels in BRAFi-resistant melanoma.

ARV as a PROTAC molecule and PTEN as a therapeutic gene stand as distinct modalities in terms of their physicochemical properties, making it challenging for co-delivery. ARV being a PROTAC class of molecule, it belongs to the Beyond Rule of 5 chemical space, exhibiting poor solubility, poor permeability, and susceptibility to rapid enzymatic degradation on administration.[16] Previously, both polymer- and lipid-based formulations of ARV have been developed for the treatment of different solid tumors.[12,17,18] However, liposomal formulations are versatile drug delivery systems given their biocompatibility, non-immunogenicity, and high drug-loading capacity. They possess several advantages with regard to drug delivery, including enhanced drug solubility and permeability, prolonged circulation of drugs, providing protection against drug degradation and serving as a sustained release system to reduce any toxic effects.[19,20] Hence, we fabricated ARV-825 loaded nanoliposomes (AL-NANOs) to incorporate this bulky molecule within its phospholipid bilayers and deliver in BRAFi-resistant melanoma models. In contrast, PTEN plasmid being an anionic macromolecule is susceptible to enzymatic degradation and rapid clearance upon systemic administration. Lipid-based nanocarriers have been widely explored as non-viral vectors to deliver nucleic acids in form of plasmid DNA, mRNA, siRNA or antisense oligonucleotides. Among them, lipid nanoparticles (LNPs) have recently gained traction due to their attractive properties, including non-immunogenicity, ease of manufacture and scale-up, larger payloads, and flexibility of design.[21] Several LNP-based formulations are currently in the market given their clinical success in oncology as well as viral infections. Patisiran (Onpattro) developed by Alnylam Pharmaceuticals was the first-in-human LNP-based small interfering RNA (siRNA) drug that received US FDA approval in 2018 for the treatment of polyneuropathy in people with hereditary transthyretin-mediated amyloidosis.[22] Two LNP-formulated mRNA-based vaccines developed by Moderna and Pfizer/BioNTech to deliver spike mRNA were also approved by the US FDA in 2020 for the prevention of coronavirus disease 2019 (COVID-19).[23] In this context, we prepared PTEN plasmid DNA loaded LNPs (PL-NANOs) by microfluidic mixing for its successful delivery in BRAFi-resistant melanoma cells.

Lipid-based nanocarriers have proven to be among the most successful approaches for achieving drug and gene co-delivery efficiency in cancer therapy.[24] Hence, the main objective of this research was to develop and characterize both AL-NANO and PL-NANO for simultaneous delivery of ARV and PTEN plasmid, respectively, in two-dimensional (2D) and three-dimensional (3D) cell culture models of BRAFi-resistant melanoma and identify their synergism *in vitro*. This is the very first report illustrating concomitant delivery of a lethal combination of BRD4 PROTAC and a tumor suppressor gene to cooperatively inhibit the progression of drug-resistant melanoma.

RESULTS

Combination of ARV and PTEN is synergistic in BRAFi-resistant melanoma

Primarily, we analyzed the synergism between ARV and PTEN plasmid to achieve enhanced therapeutic potency in BRAFi-resistant melanoma. For this purpose, we transfected PTEN plasmid using a standard transfection reagent transit LT1 in both acquired (A375V) and intrinsically (RPMI-7951) resistant melanoma cells. PTEN plasmid alone delivered at 250 ng per well resulted in 37.5 ± 2.2% and 46.8 ± 4.3% cell kill in A375V and RPMI-7951 cell lines, respectively. To test the effect of simultaneous delivery of ARV and PTEN in BRAFi-resistant melanoma, we treated both A375V and RPMI-7951 cell lines with these therapeutic agents alone and in combination. Significant synergism was observed between ARV and PTEN, as seen in Figure S5. Clearly, transfection with

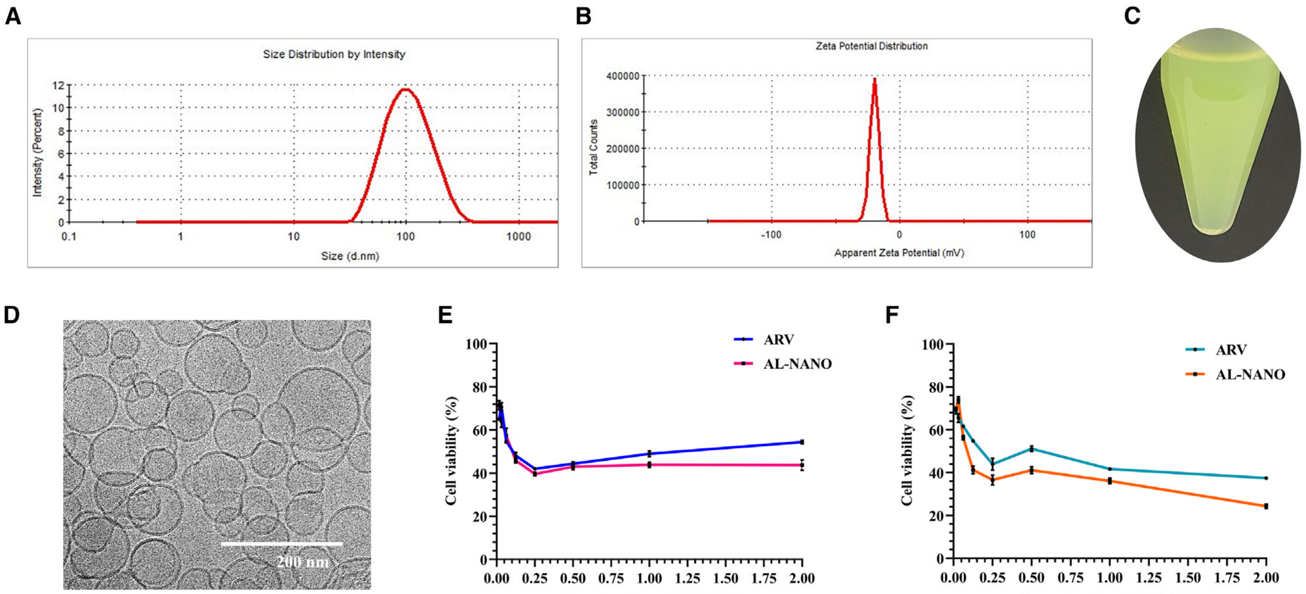

Figure 1. Physicochemical characteristics and anticancer activity of AL-NANO in VEM-resistant melanoma cell lines

DLS graphs illustrating (A) unimodal particle size distribution and (B) neutral zeta potential of AL-NANO. (C) Image of formulated AL-NANO depicting a clear and stable ARV-loaded nanoformulation. (D) Cryo-TEM image of AL-NANO reveals spherical unilamellar liposomal vesicles in the size range of 80–100 nm (n = 3, data are presented as mean ± SD). MTT cytotoxicity curves in (E) A375V and (F) RPMI-7951 cell lines indicating anticancer potency of ARV as solution and liposomal formulation in acquired and intrinsically BRAFi-resistant melanoma cell lines. Control: non-treated cells (n = 6, data are presented as mean ± SD). Scale bar, 200 nm.

PTEN plasmid substantially improved the cytotoxic effect of ARV as shown by fold-reduction in its half-maximal inhibitory concentration (IC_{50}) in both the resistant cell lines (Table S1). Combination with PTEN resulted in approximately 8-fold decrease in the IC_{50} of ARV in A375V cells, while it demonstrated about a 15-fold decrease in the IC_{50} of ARV in RPMI-7951 cells, leading to an IC_{50} value of as low as 10 nM.

Physicochemical characterization and *in vitro* anticancer activity of AL-NANO

Modified hydration method resulted in stable ARV-loaded nanoliposomes (AL-NANOs) depicting a particle size of 87.11 ± 5.72 nm, polydispersity index of 0.203 ± 0.08, and nearly neutral zeta potential of −19.82 ± 3.65 mV as seen in the dynamic light scattering (DLS) graphs depicted in Figures 1A and 1B. More important, an entrapment efficiency of greater than 99% was achieved for ARV, along with drug loading of 0.1% w/v within these nanoliposomes as evaluated by high-performance liquid chromatography (HPLC) (drug peak as shown in Figure S2). The formation of AL-NANO resulted in a clear liposomal nanosystem as seen in Figure 1C, with no signs of drug precipitation or lipid aggregation. AL-NANO liposomes predominantly appeared as unilamellar spherical vesicles as per our cryogenic transmission electron microscopy (cryo-TEM) analysis to show a highly dense lipid bilayer surrounding an aqueous core. The diameter of AL-NANO was observed to be within the range of 80–100 nm, which is consistent with the particle size distribution analysis by DLS (Figure 1D).

The *in vitro* cytotoxicity of ARV alone and in liposomal formulation AL-NANO was evaluated in A375V (acquired resistance) and RPMI-7951 (intrinsic resistance) cells. The 3-(4,5-dimethylthiazol-2-yl)-2,5-diphenyltetrazolium bromide tetrazolium reduction (MTT) assay-based cytotoxicity curves exhibited by ARV and AL-NANO in both the cell lines are as illustrated in Figures 1E and 1F. We observed potent anticancer efficacy of ARV in solution and liposomal formulation with no significant difference between both the treatment groups. ARV and AL-NANO showed comparable cytotoxicity with IC_{50} values of 100 ± 20 nM and 100 ± 15 nM in A375V cells, respectively, while it was 190 ± 10 nM and 125 ± 20 nM in RPMI-7951 cells, respectively.

Physicochemical characterization and *in vitro* tumor growth suppression by PL-NANO

Microfluidic mixing approach was used to prepare PTEN-pDNA-loaded ionizable LNPs at previously established and optimized processing conditions.[25] Entrapment of PTEN-pDNA in SM-102 ionizable lipid based LNPs at a 50 μg/mL concentration resulted in fabrication of PL-NANO. PL-NANO exhibited an average size of 88.27 ± 3.8 nm and PDI of 0.103 ± 0.012 with positive surface charge of +45 ± 1.8 mV before dialysis. This could be due to the addition of ionizable lipid SM-102 for successful encapsulation of PTEN-pDNA within its hydrophilic core. The PTEN plasmid solution was prepared at an acidic pH of 4.0 using a sodium acetate buffer during preparation of PL-NANO to mediate complexation of negatively charge PTEN-pDNA with protonated SM-102 ionizable lipid in acidic

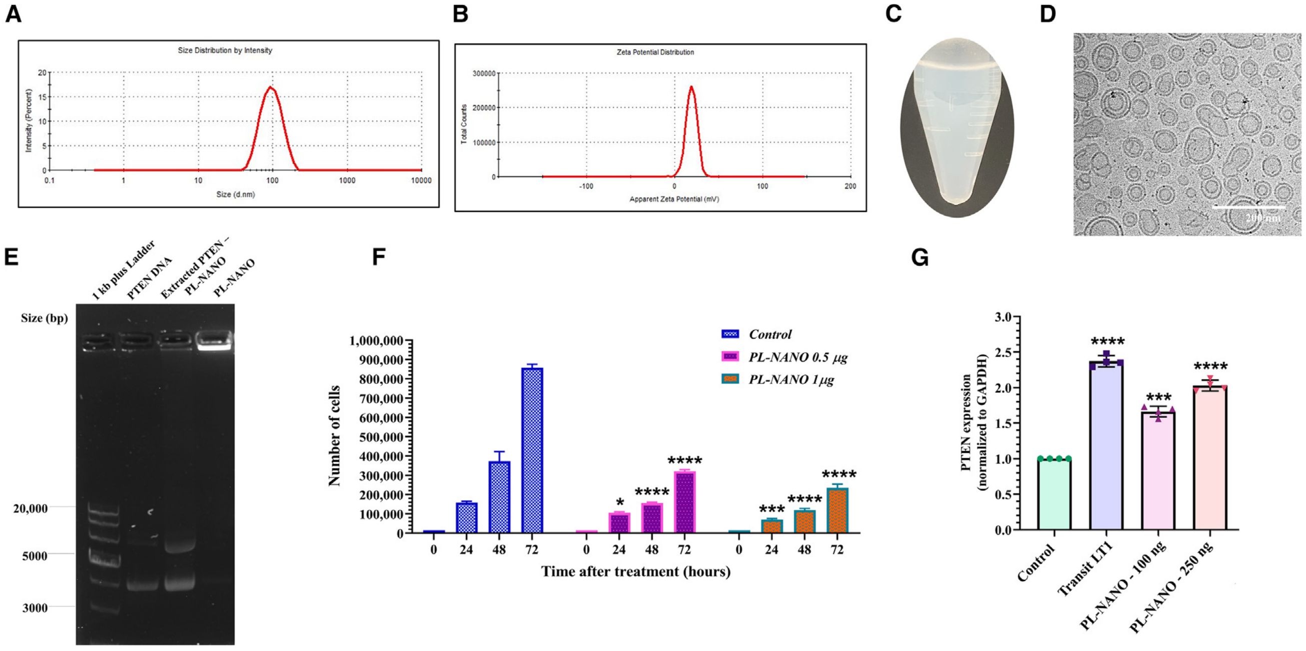

Figure 2. Physicochemical characteristics and *in vitro* tumor growth inhibitory activity of PL-NANO in VEM-resistant melanoma cell lines

DLS graphs illustrating (A) unimodal particle size distribution and (B) neutral zeta potential of PL-NANO. (C) Image of fabricated PL-NANO depicting a blue tinge in the developed nanoformulation (n = 3d Data are presented as mean ± SD). (D) Cryo-TEM image of PL-NANO reveals spherical unilamellar liposomal vesicles in the size range of 80–100 nm. (E) Representative agarose gel electrophoresis image illustrating binding efficiency and complete entrapment of PTEN plasmid to PL-NANO. (F) Cell counting assay results depicting inhibition in growth of A375V cells by PL-NANO at different time points as compared with controls. (G) Quantitative analysis of PTEN expression following transfection with PL-NANO at different doses and Transit LT1 after 48 h of treatment in A375V cells. Significant difference depicted in the graph is in comparison with controls. Control: non-treated cells (n = 6, data are presented as mean ± SD). *$p < 0.05$, ***$p < 0.001$, ****$p < 0.0001$.

condition. Therefore, after formation of PL-NANO, they were dialyzed against PBS pH 7.4 to remove ethanol and neutralize its surface charge. Dialysis had no significant effect on particle size of PL-NANO; however, their surface charge reduced to +19.2 ± 0.9 mV, rendering them neutral as evident from DLS graphs illustrated in Figures 2A and 2B. PicoGreen assay revealed 99.42 ± 0.08% entrapment efficiency of PTEN-pDNA within PL-NANO. Formation of PL-NANO resulted in a nanosystem exhibiting a blue tinge as clearly seen in Figure 2C. As per our cryo-TEM results, we found that PL-NANO exhibited spherical structures with mostly single lipid bilayer, depicting a size distribution between 75 and 100 nm. Similar results were obtained for DLS measurements. Some multilamellar structures were also seen due to formation of blebs, which are a result of aqueous pockets containing pDNA within the LNP structure. An outer boundary surrounding the LNPs was observed that depicts the hydration layer formed by the pegylated (PEG) lipid (Figure 2D). Also, agarose gel electrophoresis results confirmed complete entrapment of PTEN-pDNA by PL-NANO as no migration bands were observed for the LNP formulation and DNA band for extracted plasmid correlated with that of naked PTEN-pDNA (Figure 2E).

Tumor growth inhibition effect of PL-NANO was evaluated in A375V cells after transfection with PTEN-pDNA. As shown in Figure 2F, a cell counting assay was performed at different points in A375V cells. Treatment with PL-NANO resulted in inhibition of tumor cells growth at both 0.5- and 1-μg doses of PTEN-pDNA. A higher dose tested for PTEN-pDNA was more efficient in decreasing the growth of A375V cells at all time points. To determine that the tumor growth suppression shown by PL-NANO was a result of delivering PTEN-pDNA, we performed ELISA as the preliminary assay in A375V cells. After transfection with PL-NANO in A375V cells, its effect on PTEN expression was analyzed after 48 h of treatment. Transit LT1 was used as a standard transfection reagent to attain target PTEN expression. Our results indicated that transfection with PL-NANO at 100 ng and 250 ng of PTEN-pDNA led to 1.7- and 2-fold increases in PTEN expression when compared with controls. Therefore, an even lower dose of PTEN was sufficient to induce higher PTEN expression levels after transfection with PL-NANO. Moreover, when PL-NANO were transfected at 250 ng of PTEN-pDNA per well, the resultant PTEN expression levels were comparable with that of positive control transit LT1, which induced about a 2.4-fold increase in PTEN expression when transfected at the same PTEN plasmid dose (Figure 2G). Therefore, these results indicate successful delivery of PTEN-pDNA by formulated PL-NANO to significantly inhibit the growth of BRAF-resistant A375V melanoma cells at tested doses.

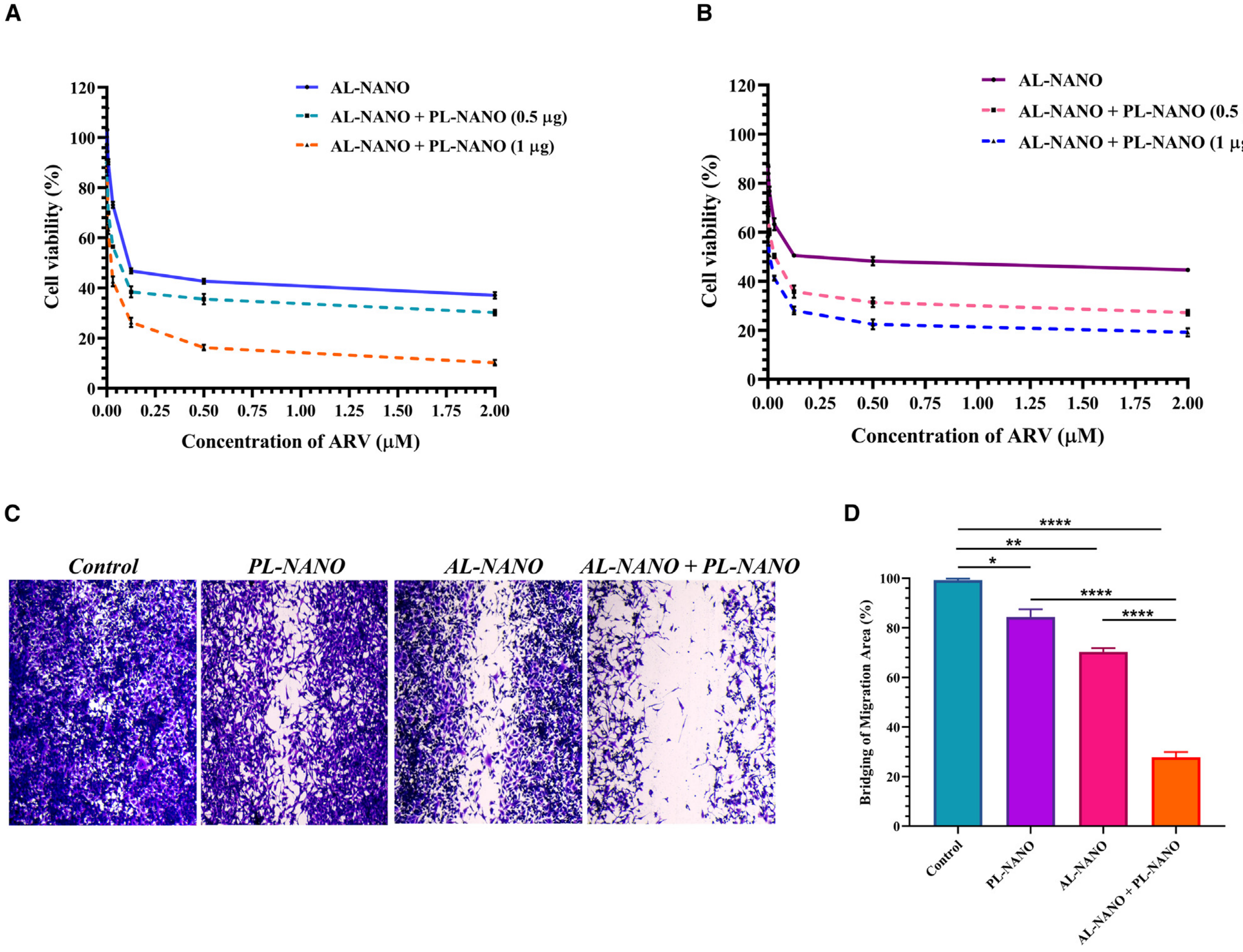

Figure 3. *In vitro* cell viability and migration assay of AL-NANO and of PL-NANO in VEM-resistant melanoma cell lines

MTT cytotoxicity curves in (A) A375V and (B) RPMI-7951 cell lines indicating strong synergism between AL-NANO and PL-NANO in acquired and intrinsically BRAFi-resistant melanoma cell lines. (C) Microscopic images of scratch assay of PL-NANO and AL-NANO treated cells alone and in combination as compared with control. (D) Percentage inhibition of migration produced by PL-NANO, AL-NANO, and PL-NANO + AL-NANO in A375V cells. Control: non-treated cells ($n = 6$, data are presented as mean ± SD). $^{**}p < 0.01$, $^{****}p < 0.0001$.

Synergism between AL-NANO and PL-NANO in 2D cell culture models of BRAFi-resistant melanoma

Cell viability assay

To identify the synergism between AL-NANO and PL-NANO, an MTT cytotoxicity assay was performed. For this, A375V and RPMI-7951 cells were initially transfected with PL-NANO at 0.5 and 1 μg PTEN-pDNA per well for 4 h followed by treatment with AL-NANO at different concentrations. The cytotoxic effects of proposed combination were compared with that of AL-NANO alone. As shown in Figures 3A and 3B, combination treatment resulted in significantly enhanced cytotoxicity of AL-NANO at both the tested doses of PL-NANO. PL-NANO when transfected at a dose of 0.5 μg, decreased the IC_{50} of ARV by 2-fold in A375V cells while that by 4-fold in RPMI-7951 cells, as illustrated in Table 1. Certainly, a higher dose of PL-NANO (1 μg) exhibited even higher potency with 5.2-fold and 14.4-fold decrease in the IC_{50} of AL-NANO in A375V and RPMI-7951 cells, respectively. An IC_{50} of as low as 20 nM was achieved in A375V cells and 8 nM was obtained in RPMI-7951 cells for AL-NANO when transfected with a higher dose of PL-NANO.

In vitro *migration assay*

Cell migration is a key procedure involved in the progression of cancer growth; therefore, an *in vitro* scratch assay was performed to study the cell migration behavior of A375V cells and effect of various treatment groups alone or in combination *in vitro*. Figure 3C elucidates the scratch area of A375V cells treated with PL-NANO (250 ng PTEN-pDNA), AL-NANO (10 nM ARV), and PL-NANO + AL-NANO (250 ng PTEN-pDNA/10 nM ARV) as compared with the control group. Within a period of 24 h, we observed that the control group exhibited nearly 99% bridging of scratch area in A375V cells,

Table 1. IC_{50} values of ARV in AL-NANO alone and in combination with PL-NANO in BRAFi-resistant melanoma cell lines (n = 6, data are presented as mean ± SD)

Cell line	Amount of PTEN plasmid in PL-NANO (μg)	IC_{50} of ARV (μM)		Fold decrease in IC_{50} of ARV
		AL-NANO	AL-NANO + PL-NANO	
A375V	0.5	0.11 ± 0.07	0.05 ± 0.01	∼2.0-fold
	1	0.11 ± 0.07	0.02 ± 0.002	∼5.2-fold
RPMI-7951	0.5	0.12 ± 0.05	0.03 ± 0.005	∼4.0-fold
	1	0.12 ± 0.05	0.008 ± 0.001	∼14.4-fold

demonstrating the highly aggressive behavior of this acquired BRAFi-resistant cell line. Transfection with PL-NANO led to nearly 78% bridging of scratch area and AL-NANO treatment led to about 70% bridging of scratch area at tested concentrations. However, combination treatment of PL-NANO and AL-NANO was successful in significantly inhibiting the proliferation and migration of A375V cells in comparison with individual treatment groups, leading to about 25% bridging of the scratch area, as shown in Figure 3D.

Clonogenic survival assay

The clonogenic cell survival assay determines the ability of a cancer cell to proliferate indeterminately, thereby retaining its reproductive ability to form a large colony. It is a frequently performed assay to quantify reproductive cell survival *in vitro*. We identified the ability of A375V cells to form colonies after treatment with PL-NANO and AL-NANO alone, as well as in combination at a dose of 250 ng of PTEN-pDNA and 10 nM of ARV. As clearly depicted in Figure 4, A375V cells treated with PL-NANO did not result in any significant effect in terms of number or size of colonies formed while AL-NANO treated cells showed a significant decrease in the number of colonies formed, while their size was comparable with that of the control group. Importantly, combination of PL-NANO and AL-NANO showed a substantial decrease in the number as well as size of colonies formed in comparison with control or individual treatment groups. Also, combination group formed approximately 5-fold lower number of colonies when compared with control group while approximately 4-fold and approximately 2-fold lower number of colonies were observed in comparison to individual PL-NANO- and AL-NANO-treated groups. In terms of size of colonies formed, combination treatment resulted in a 5- to 6-fold smaller size of colonies as compared with control or individual treatment groups in A375V cells.

Vasculogenic mimicry assay

We identified the potential of PL-NANO (250 ng of PTEN-pDNA) and AL-NANO (25 nM ARV) in inhibiting the vasculogenic mimicry (VM) shown by A375V cells. The extensive VM tube formation seen in control group of A375V cells embedded on the matrix gel reveals a sign of tumor progression and metastasis *in vitro* (Figure 5A). Whereas treatment with PL-NANO and AL-NANO individually showed inhibition of VM channel formation, the combination group was found to inhibit the VM channel formation further drastically in A375V cells. As seen in Figure 5B, treatment with PL-NANO decreased the branching points by 20%, while AL-NANO treatment decreased the number of branching points by 30% as compared with controls. More important, PL-NANO and AL-NANO combination treatment was the most effective in significantly decreasing the number of branching points formed by A375V cells by 75%–80% to illustrate the inhibition of VM channel formation.

Flow cytometry for in vitro *apoptosis analysis*

Apoptosis is a type of programmed cell death in which a series of molecular steps lead to the death of cancer cells. To identify the apoptosis-mediated cell death mechanism of both the nanoformulations, we performed the annexin V apoptosis assay in both acquired and intrinsically VEM-resistant melanoma cell lines on exposure to PL-NANO (500 ng of PTEN-pDNA) and AL-NANO (50 nM ARV) alone and in combination. As shown in Figure 5C, PL-NANO and AL-NANO treatment resulted in 11.8% and 18.4% apoptosis in A375V cells, while their combination showed significantly higher amount of apoptosis (31.9%) as compared with individual groups or control. Similarly, an apoptosis assay in RPMI-7951 cells revealed 11.9% and 13% apoptosis mediated by PL-NANO and AL-NANO, respectively, while their combination exhibited a significantly higher apoptotic population of 30.5% (Figure 5D). These findings showed that the antiproliferative activity of PL-NANO and AL-NANO alone as well as in combination was exerted through induction of apoptosis in BRAFi-resistant melanoma cell lines.

Synergism between AL-NANO and PL-NANO in 3D multicellular tumor spheroids of BRAFi-resistant melanoma

Given the advantage of using 3D multicellular tumor spheroids (MCTSs) to closely mimic the *in vivo* tumor microenvironment, we screened the anticancer efficacy of PL-NANO and AL-NANO alone and as combination treatment in both acquired and inherently BRAFi-resistant melanoma spheroids. According to the bright field images depicting A375V spheroids exposed to different treatments for 5 days as shown in Figure 6A, the growth of control spheroids was found to be very aggressive. In the absence of any treatment, spheroids belonging to the control group exhibited an increase in their area by more than 10-fold in merely 5 days of incubation in complete DMEM as clearly seen in Figure 6B. Since PTEN plasmid encapsulated in PL-NANO acts by inhibiting the *in vitro* tumor growth, a similar trend was observed in the 3D MCTS. PL-NANO-treated spheroids demonstrated sustained growth while maintaining the area of spheroids to be significantly lower than that of controls. While ARV is a BRD4 degrader with potent anticancer effects in

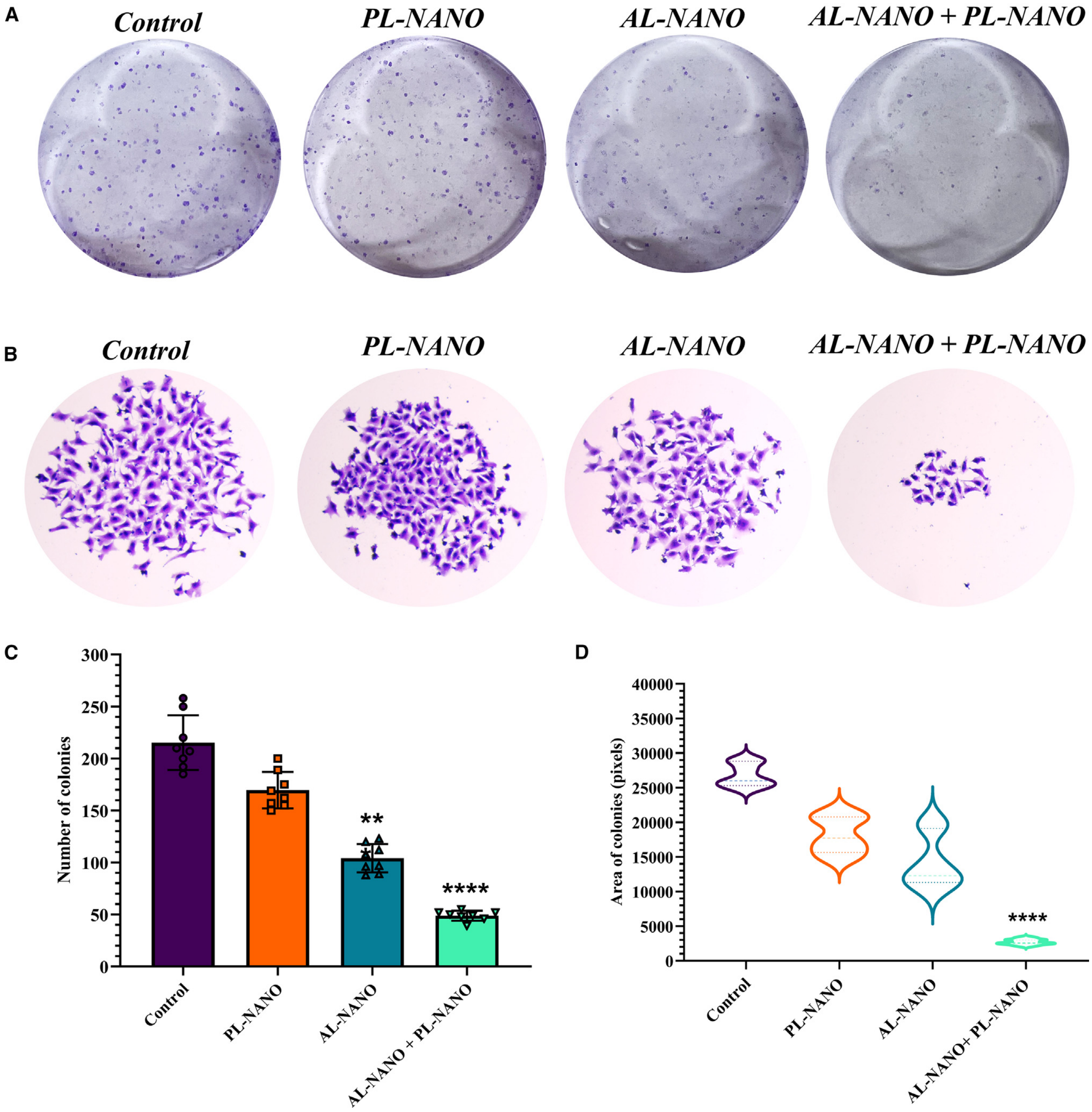

Figure 4. Effect of treatment with PL-NANO and AL-NANO alone and in combination on the colony forming ability of A375V BRAFi-resistant melanoma cells
(A) Representative images of colonies formed by A375V cells following crystal violet staining for individual and combination treated cells. (B) Images depicting size of colonies formed for each treatment and control groups. (C) Quantitative illustration of percentage decrease in the number of colonies formed with individual and combination treatment as comparison to control. (D) Quantitative analysis of size of colonies present in each treatment group as compared with control. Control: non-treated cells ($n = 6$, data are presented as mean ± SD). $^{**}p < 0.01$, $^{****}p < 0.0001$.

BRAFi-resistant melanoma, AL-NANO-treated spheroids showed a gradual decrease in their diameter and area when compared with PL-NANO-treated and control spheroids. AL-NANO was found to be more effective in inhibiting the growth of spheroids than PL-NANO. However, PL-NANO and AL-NANO combination-treated spheroids were found to exhibit even more substantial inhibition of tumor growth when compared with individual treatments or control groups. The area of combination treated spheroids on day 5 was

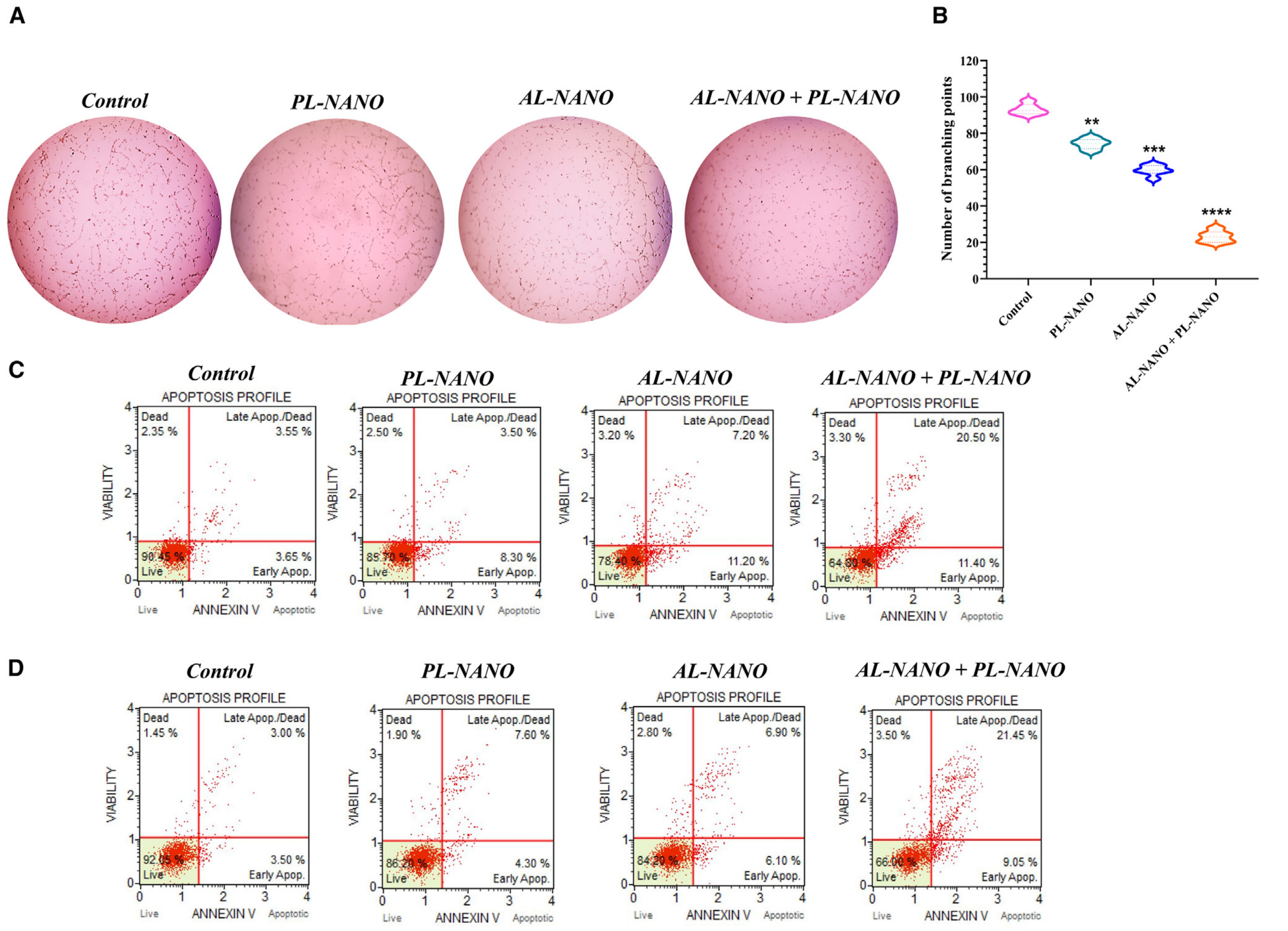

Figure 5. Evaluating the effect of PL-NANO and AL-NANO treatment alone and in combination on VM and *in vitro* apoptosis exhibited by acquired and intrinsically BRAFi-resistant melanoma cells

(A) VM images of A375V cells treated with PL-NANO and AL-NANO as individual or combination treatment. (B) Quantitative analysis of number of branching points formed in each treatment group as compared with control. (C) Significant apoptotic effect of PL-NANO and AL-NANO combination resulting in 31.9% apoptotic cell population in A375V cells. (D) Substantial apoptosis by PL-NANO and AL-NANO combination resulting in 30.5% apoptotic cell population in RPMI-7951 cells. Control: non-treated cells (n = 6, data are presented as mean ± SD). $^{**}p < 0.01$, $^{***}p < 0.001$, $^{****}p < 0.0001$.

found to be comparable with that on day 0, illustrating complete tumor growth inhibition of 3D MCTS. The area of spheroids for PL-NANO and AL-NANO alone treatments was yet significantly lower than that of controls on day 5. Also, the morphology of 3D MCTS exposed to different treatments was distinct from the control group. In the case of control spheroids, they grew with time to show a regular and smooth surface with a dark and dense core depicting the necrotic and hypoxic regions. The proliferating region was found to be highest in control spheroids, indicating its high growth rate during the period of 5 days. A similar smooth surface was observed for spheroids treated with PL-NANO, however with significantly inhibited growth of their proliferation region. In the case of AL-NANO-treated spheroids, an irregular surface was observed with even smaller proliferation region given the cell kill exhibited by ARV. In the combination treated group, the area of spheroids was the smallest with substantial inhibition in growth of both proliferation and hypoxic regions of spheroids. The surface of these spheroids was also found to be rough and irregular with higher apoptotic population present on the periphery of spheroids. The viability of cells within the A375V spheroids was determined using Live/Dead Cell Assay Kit and analyzed by fluorescence microscopy. Red fluorescence within spheroids is produced by the ethidium homodimer-1 stain indicative of apoptotic or dead cells, green fluorescence denotes live cells stained by calcein AM, and blue fluorescence is exhibited by nuclei of cells as stained by DAPI. Figure 6C shows that control spheroids exhibit strong green fluorescence of rapidly proliferating live cells especially on the periphery. While PL-NANO-treated spheroids exhibited red fluorescence toward the hypoxic region, some green fluorescence on their periphery. However, AL-NANO-treated spheroids showed higher red fluorescence, indicative of apoptotic or dead cells, while the combination treated

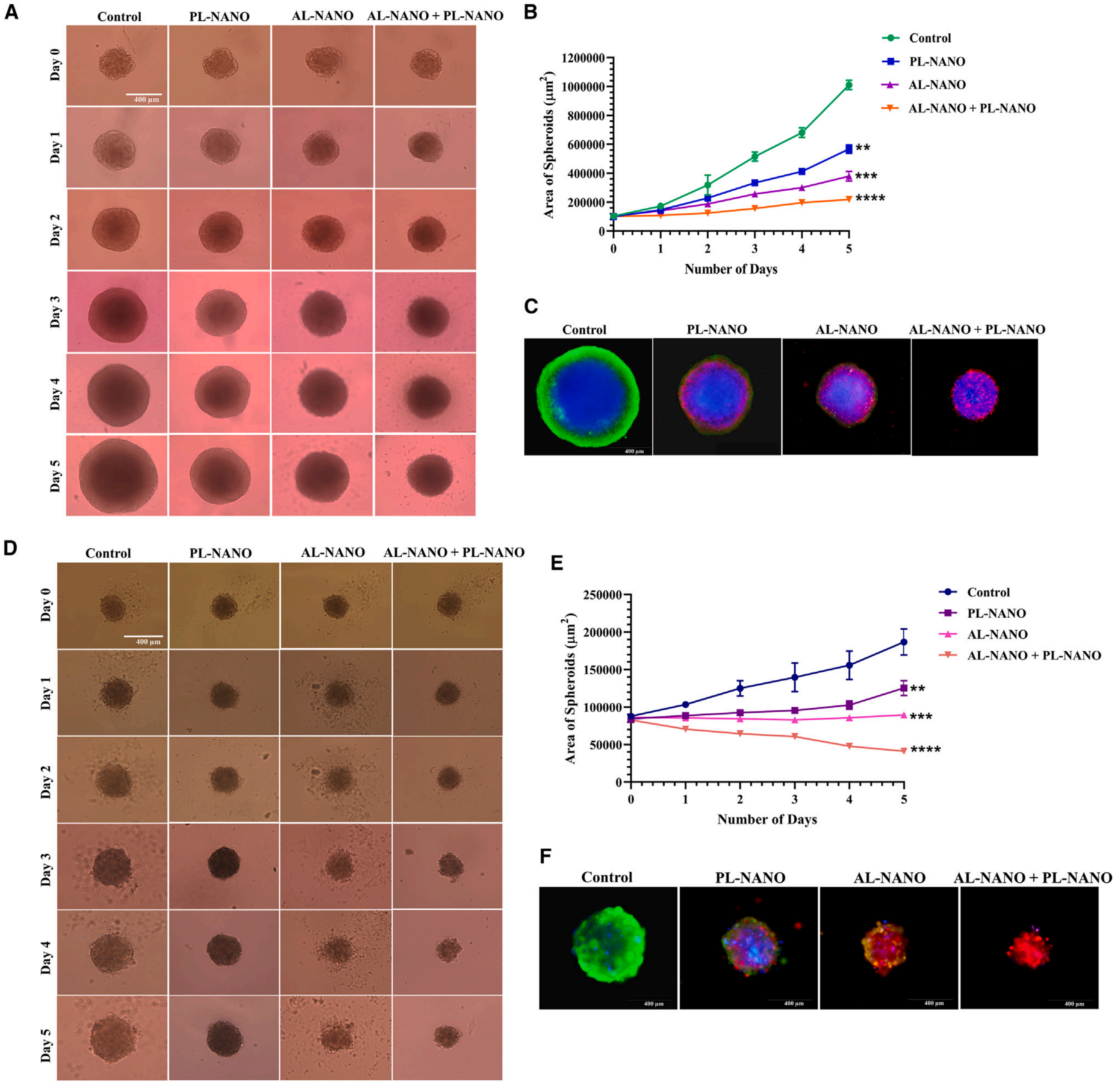

Figure 6. Results for cell viability within A375V and RPMI-7951 3D MCTSs following treatment with PL-NANO and AL-NANO alone and in combination

(A) Representative bright field images of A375V 3D MCTS treated with PL-NANO, AL-NANO, and PL-NANO + AL-NANO during 5 days of treatment. A significantly higher reduction in size of A375V 3D MCTS was observed for combination group in comparison to individual treatments or control. (B) Comparison of the area of A375V 3D MCTS treated with different groups during 5 days of treatment. Significant difference was observed in the area of spheroids treated with PL-NANO, AL-NANO and PL-NANO + AL-NANO in comparison with controls. (C) Fluorescence images signifying apoptosis of A375V 3D MCTS treated with various groups. (D) Representative bright field images of RPMI-795 3D MCTS treated with PL-NANO, AL-NANO, and PL-NANO + AL-NANO during 5 days of treatment. A significantly higher reduction in size of RPMI-7951 3D MCTS was observed for combination group in comparison to individual treatments or control. (E) Comparison of the area of RPMI-7951 3D MCTS treated with different groups during 5 days of treatment. Significant difference was observed in the area of spheroids treated with PL-NANO, AL-NANO, and PL-NANO + AL-NANO in comparison with control. (F) Fluorescence images signifying apoptosis of RPMI-7951 3D MCTS treated with various groups. Composite images of DAPI (blue), Calcein AM (green) and ethidium homodimer-1 (red). Control: non-treated cells (n = 6, data are presented as mean ± SD). $^{**}p < 0.01$, $^{***}p < 0.001$, $^{****}p < 0.0001$. Scale bars, 400 µm.

group displayed even higher and bright red fluorescence denoting a higher population of apoptotic and dead cells when compared with individual treatment groups or controls. These results were indicative of the higher cytotoxicity associated with a proposed combination group in A375V spheroids.

Similar results were observed for combination treatment in inherently BRAFi-resistant RPMI-7951 spheroids as shown in Figure 6D. The growth rate of the control RPMI-7951 spheroids was slower as compared with A375V spheroids. However, treatment with PL-NANO inhibited their proliferation, as seen by smaller area of spheroids with darker hypoxic core. AL-NANO was more potent in completely inhibiting the growth of tumor spheroids during 5 days of treatment. In contrast, PL-NANO and AL-NANO combination-treated 3D MCTS showed reduction in area of spheroids with time indicating their higher anticancer effects (Figure 6E). The hypoxic or necrotic regions could not be seen clearly in RPMI-7951 3D spheroids given their smaller size than A375V spheroids. However, the treatment groups could successfully inhibit the proliferation region of these spheroids with maximum inhibitory effects observed in the combination group. The fluorescence images of RPMI-7951 spheroids showed maximum bright red fluorescence in combination treated group further signifying its apoptotic effect as compared with individual treatments or control groups (Figure 6F).

Western blot analysis

A western blot analysis was performed to identify the effect of treatment with PL-NANO and AL-NANO on their target pathways in BRAFi-resistant melanoma. Our results revealed successful delivery of PTEN gene in A375V cells as observed by significantly upregulated expression levels of PTEN in PL-NANO and combination treated groups. Following increased levels of PTEN, we also observed substantially reduced levels of pMAPK/MAPK after treatment with PL-NANO and combination since PTEN counteracts the RAS/MAPK signaling pathway as a protein phosphatase, while in AL-NANO and combination treated groups, significant downregulation of BRD4 protein and subsequently c-Myc oncogene was observed. As a selective BRD4 PROTAC, ARV in AL-NANO and combination was successful in attaining degradation and downregulation of BRD4 target protein. As the pathways regulated by both PTEN and ARV converge toward downregulation of c-Myc, our results indicated substantially higher downregulation of oncogenic c-Myc expression levels by the combination treated group in A375V cells (Figures 7A and 7B).

Biocompatibility and systemic safety of AL-NANO and PL-NANO

Madin-Darby canine kidney (MDCK) cells monolayer was used as an epithelial cell model to identify any toxicity associated with PL-NANO and AL-NANO after 24 h of treatment. Transfection with PL-NANO did not cause any toxicity in MDCK cells monolayer, resulting in a cell viability of 95 ± 2.3% after 24 h of treatment. We also observed negligible toxicity after treatment with AL-NANO after 24 h in the MDCK cell monolayer as shown in Figure 7C. Moreover, combination treatment with PL-NANO and AL-NANO resulted in greater than 80% cell viability at all tested concentrations of ARV in MDCK cells, indicating the biosafety of both lipid nanoformulations.

Our *in vitro* hemolysis assay results indicated negligible hemolysis (<5%) by PL-NANO, even at the 200 μM concentration of ionizable lipid SM-102 in formulated LNPs. Also, AL-NANO was found to be hemocompatible (<5% hemolysis) even at concentration of 10 μg/mL ARV, as compared with the positive control (sodium lauryl sulfate), which showed complete hemolysis of mice red blood cells (RBCs). Furthermore, we observed immediate and complete redispersion of RBCs after centrifugation, implying that neither PL-NANO nor AL-NANO modified the surface characteristics of RBCs, as seen in Figure 7D.

Stability analysis of AL-NANO and PL-NANO

The AL-NANO and PL-NANO formulations were subjected to a stability study in liquid form for a period of 3 months at 4°C. As depicted in Figure S6A, AL-NANO were found to be stable in terms of their physicochemical characterization. There was no statistically significant difference observed in the particle size, polydispersity index, or zeta potential of AL-NANO after 3 months as compared with freshly prepared formulation. Also, ARV was found to be completely encapsulated within the liposomal lipid bilayer of AL-NANO, preventing its precipitation or physicochemical degradation. The encapsulation efficiency of ARV was found to be greater than 95% in AL-NANO at the end of 3 months (Figure S6B). PL-NANO also did not show any significant change in its particle size or zeta potential during its storage, with no signs of precipitation observed (Figure S6C). Moreover, the integrity of PTEN-pDNA was preserved with complete entrapment in PL-NANO following 3 months as depicted by agarose gel electrophoresis results (Figure S6D).

DISCUSSION

Oncogenic c-Myc is a well-established driver of tumor initiation and maintenance, and is often allied with all the hallmark characteristics of various solid tumors, including malignant melanoma.[26] Given this critical role of undruggable c-Myc oncoprotein, researchers have attempted to directly target c-Myc resulting in inhibitors and degraders that show suboptimal pharmacological properties, low *in vivo* potency, or unacceptable off-target effects limiting their clinical application.[27–30] Therefore, our approach was to use ARV as a BRD4 selective PROTAC and PTEN plasmid as a tumor suppressor gene for simultaneous indirect inhibition of c-Myc expression in VEM-resistant melanoma. Our preliminary cytotoxicity assay results demonstrated strong synergism between ARV and PTEN plasmid when delivered through a standard transfection reagent (LT1) in both acquired and intrinsically VEM-resistant melanoma cell lines, to support the hypothesis of this work.

Combination therapy stands as a common standard first-line treatment approach for several malignancies that have resulted in improved clinical outcomes. Integrating anticancer drugs and genes has proven to enhance the synergistic action and increase the

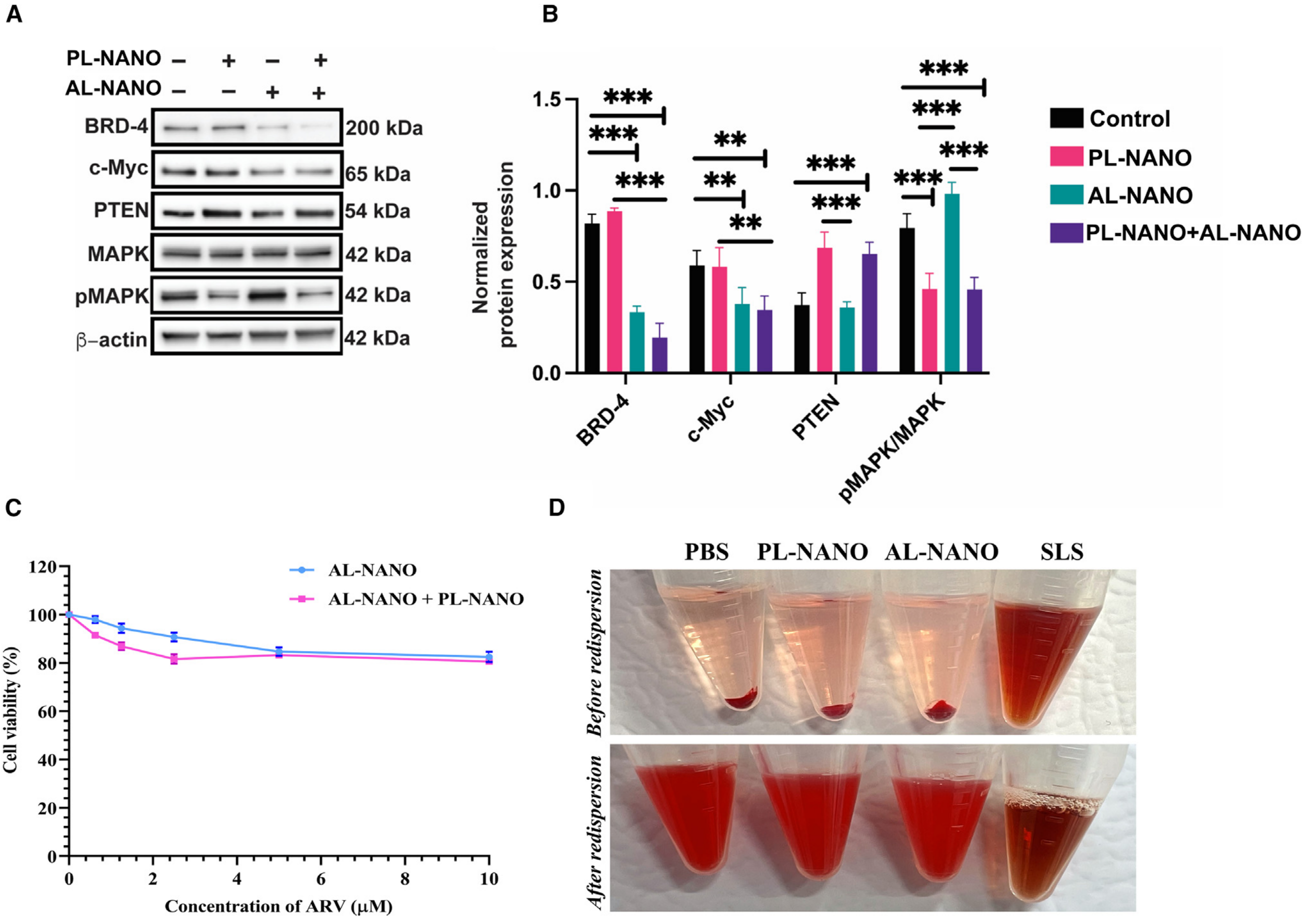

Figure 7. Results for target protein expression, biocompatibility and *in vitro* hemolysis analysis for PL-NANO and AL-NANO

(A) Representative western blots for expression of BRD-4, c-Myc, PTEN, MAPK, and pMAPK proteins in A375V cells are shown. β-Actin was used as an internal control. (B) Quantification of the western blot results for expression of each target protein in A375V cells. (C) The cytotoxicity curve represents negligible toxicity exhibited by PL-NANO and AL-NANO both alone and in combination in MDCK cells. PL-NANO alone resulted in more than 95% cell viability when tested in MDCK cells at a dose of 500 ng per well. Control: non-treated cells ($n = 6$, data are presented as mean ± SD). (D) Negligible hemolysis demonstrated by PL-NANO at 200 μM concentration of ionizable lipid SM-102 as well as AL-NANO at concentration of 10 μg/mL ARV as tested in mice RBCs. $^{**}p < 0.01$, $^{***}p < 0.001$.

efficiency of overcoming drug resistance in multiple tumors.[31] Lipid-based nanocarriers are among the most successful nanotechnology-based approaches that have been used for simultaneous drug and gene delivery given their versatile nature, high drug loading efficiency, and biocompatibility. For instance, Swami et al.[32] developed a systemically stable DTX-lipoplex system to co-deliver DTX and SIRT1-shRNA for treatment of breast cancer. Also, Younis et al.[33] formulated ultra-small LNPs to co-deliver sorafenib (SOR) and MK-siRNA for the treatment of SOR-resistant hepatic carcinoma, which showed high *in vivo* anticancer potential at a low dose of individual therapeutic agents. However, PROTAC molecules and plasmid genes are extremely versatile in terms of their physicochemical properties. ARV is a large hydrophobic agent with poor solubility and permeability, while PTEN plasmid is an anionic macromolecule susceptible to enzymatic degradation on systemic administration. It would be challenging to incorporate both these moieties into a single lipid-based nanocarrier with a high loading capacity while maintaining their therapeutic activity. Thus, we designed two different lipid nanocarriers for each of these therapeutic agents to explore their synergism in 2D and 3D BRAFi-resistant melanoma models following their simultaneous delivery. In terms of gene delivery via LNPs, a key component of this formulation is the cationic or ionizable lipid that is responsible for negatively charged nucleic acid complexation and its endosomal escape for subsequent cytosolic delivery. New generations of ionizable lipids have been synthesized to pose certain advantages like ionizable pH-sensitive tertiary amine groups to avoid systemic toxicity, addition of unsaturated bonds in hydrophobic lipid chain to enhance membrane fusion, and endosomal escape capability, as well as incorporation of ester bonds to attain biodegradability. Examples of these ionizable lipids that are present in US FDA-approved formulations include Dlin-MC3-DMA (Onpattro), ALC-0315 (Pfizer/BioNTech COVID-19 vaccine), and SM-102

(Pfizer/BioNTech COVID-19 vaccine). Therefore, we screened some of the mentioned ionizable lipids in addition to other first generation ionizable and cationic lipids in our previous work to identify SM-102 as the most suitable complexing head group for PTEN plasmid delivery.[25] PL-NANO were formulated using microfluidic mixing to achieve a homogeneous nanoparticle dispersion with neutral surface charge and greater than 99% entrapment efficiency of PTEN-pDNA. PL-NANO majorly exhibited single bilayer containing nanoparticles with a small population of multilamellar structures that could be a result of internal defects or bleb formation within LNPs. Such structural defects could be due to PTEN-pDNA being separated from the lipid core. The outer shell visualized is formed by the PEG lipid that forms a hydration layer around the LNPs. Our results are in accordance with the previously established literature where similar observations have been made for mRNA-loaded ionizable LNPs.[34,35] It has been proven that there is a downregulation of PTEN gene expression following acquired resistance against BRAFi in melanoma.[13] Therefore, delivering PTEN plasmid via PL-NANO resulted in significant tumor growth inhibition in A375V cells. In contrast, RPMI-7951 is a PTEN-deleted melanoma cell line. Deletion of PTEN in this cell line contributes to its intrinsic mutations responsible for inherent resistance against BRAFi. Hence, delivering PTEN plasmid in RPMI-7951 cells resulted in nearly 50% cell growth inhibition by PL-NANO.

We chose liposomes as the lipid-based drug delivery system to entrap ARV and simultaneously deliver with PL-NANO for assessment of their synergism. Liposomes tend to possess a higher drug loading capacity and are biocompatible in nature, portraying a safe and effective lipid nanocarrier for ARV delivery. Also, liposomes possess a lipid bilayer surrounding an aqueous pocket, which could be more effective in entrapping a large molecule like ARV as a stable nanoformulation. Liposomal systems have been previously used to entrap ARV alone or in combination with other small anticancer molecules to reveal complete entrapment for successful delivery in different solid tumors.[11,18] Hence, we developed ARV-loaded liposomes by using the modified hydration method. This technique has been proven to show promising stability and entrapment efficiency of anticancer molecules given that it promotes the distribution of a lipophilic drug within the inner lipid bilayer and restricts rapid diffusion of the drug to the aqueous phase.[36] Moreover, entrapment of ARV within liposomal bilayers of AL-NANO would overcome its physicochemical challenges by enhancing its solubility and cellular permeability, while maintaining its anticancer activity in BRAFi-resistant melanoma cells. Resultant AL-NANO formed a unimodal and unilamellar liposomal nanosystem with potent anticancer activity in both acquired and intrinsically VEM-resistant melanoma cells lines, and therefore was used to screen its synergistic antitumor potential with PL-NANO in BRAFi-resistant melanoma cells.

Our *in vitro* cytotoxicity assay results indicated substantial synergism between PL-NANO and AL-NANO in both acquired and intrinsically BRAFi-resistant melanoma cells. Further, combinatorial delivery of both these nanocarriers not only inhibited the migration potential but also mitigated the *in vitro* cell survival of highly proliferative BRAFi-resistant melanoma cells as compared with individual treatments. In various malignant solid tumors, including melanoma, VM is described as a distinct characteristic that involves the formation of *de novo* microvascular structures by cancer cells that allows them to generate a channel network able to provide blood supply for tumor growth.[37,38] Since these tumor cell-generated VM channels play an important role in tumor development, invasion, and metastasis independent of tumor angiogenesis, it was essential to evaluate the effect of our combination therapy on inhibition of BRAFi-resistant melanoma progression by prohibiting formation of VM channels. It has been previously reported that the metastasis of melanoma is due to the overexpression of oncogenic c-Myc, which promotes VM via the c-Myc/snail/Bax signaling pathway.[39] Given that ARV and PTEN both target downregulation of c-Myc expression in resistant melanoma, we observed substantially greater inhibition of VM tube formation by combination treatment as compared with individual treatment groups. Importantly, we observed substantial apoptosis induced by PL-NANO and AL-NANO when delivered simultaneously in 2D cell culture models of both acquired (A375V) and intrinsically (RPMI-7951) VEM-resistant melanoma cells lines, as compared with alone treatment groups.

The 3D MCTS express an intermediary complexity between 2D cell culture monolayer models and *in vivo* solid tumors. They closely resemble the *in vivo* tumor microenvironment in terms of heterogeneous architecture, cellular layered assembling, hypoxia, and internal gradients of nutrients.[40–42] Therefore, we assessed the antitumor potential of PL-NANO and AL-NANO combination therapy in developed 3D MCTS to observe potent anticancer activity in both acquired and intrinsically BRAFi-resistant 3D melanoma models. It resulted in significant inhibition in the growth of spheroids with altered morphology indicative of high apoptotic population as seen in the bright field images as well as fluorescence images captured following live/dead cell staining assay. These observations were made in both A375V and RPMI-7951 cells-based spheroids and we believe that PL-NANO and AL-NANO dual LNPs-based combination therapy would achieve an effective cytotoxic activity when tested *in vivo*.

We targeted various cellular signaling pathways in BRAFi-resistant melanoma to downregulate c-Myc expression by simultaneous delivery of PL-NANO and AL-NANO. PTEN plasmid encapsulated in PL-NANO would target the PI3K/Akt and RAS/MAPK cellular signaling cascades involved in cell growth, proliferation and survival of melanoma cells by ultimately targeting the c-Myc oncogene. As a lipid phosphatase, PTEN would counteract the PI3K/Akt pathway.[14,15] While acting as a protein phosphatase, PTEN would counteract the RAS/MAPK signaling pathway by inhibiting the expression levels of MAPK and pMAPK.[43] Given this, PL-NANO treatment led to dual targeting of both PI3K/Akt and RAS/MAPK pathways to finally suppress c-Myc levels in BRAFi-resistant A375V melanoma cells. In contrast, ARV entrapped within phospholipid bilayers of AL-NANO is a BRD4 targeted PROTAC. Therefore, treatment with AL-NANO would reduce the levels of oncogenic c-Myc via target BRD4 protein

degradation in BRAFi-resistant melanoma.[9,10] Therefore, we observed downregulated BRD4 and c-Myc levels in AL-NANO and combination treatment groups. Most importantly, we found lowest expression levels for c-Myc oncoprotein in combination treated A375V cells as compared with individual treatment groups. Our western blot analysis results therefore confirmed our hypothesis of targeting various cellular signaling pathways including PI3K/Akt, RAS/MAPK and BRD4 degradation to finally target c-Myc oncogene by simultaneous delivery of PL-NANO and AL-NANO in BRAFi-resistant melanoma. It is imperative to attain target protein expression following treatment with PL-NANO and AL-NANO without causing any toxicity associated with off-target effects. For this purpose, we analyzed the biocompatibility of both these formulations alone and in combination in non-cancerous MDCK cell line. MDCK cells tend to grow as a polarized monolayer *in vitro* and form tight junctions, where PTEN protein is localized at site of cellular projections. Therefore, these epithelial cells already express wild-type PTEN when they form a monolayer.[44,45] In contrast, MDCK cells express low levels of BRD4 protein and have often been used as an epithelial cell monolayer model to study permeability characteristics of PROTAC molecules including BRD4 targeted degraders.[46–48] We observed negligible toxicity in MDCK cells after 24 h of treatment with PL-NANO and AL-NANO, alone as well as in combination. These results indicate the biocompatibility of both PL-NANO and AL-NANO lipid-based formulations with no off-target effects.

Since both PL-NANO and AL-NANO were formulated for potential simultaneous delivery via parenteral administration, it was essential to analyze their systemic safety. The *in vitro* hemolysis assay evaluates hemoglobin release in the plasma as an indicator of RBC lysis following exposure to any therapeutic agent. There are abundant factors accountable for the hemocompatibility of lipid-based nanoformulations including their surface charge, size, shape, and surface modification.[49,50] In case of PL-NANO, it consisted of an ionizable head group SM-102, which renders a neutral surface charge in systemic circulation while being highly positively charged in acidic conditions. Therefore, it was crucial to identify any potential hemolytic effects associated with this property of ionizable LNPs, while it was also necessary to analyze if the surface of developed nanoliposomes AL-NANO would be toxic to the membrane of RBCs on systemic administration. For this purpose, the effect of PL-NANO and AL-NANO on mice RBCs was evaluated by performing the *in vitro* hemolysis assay. We observed no signs of hemolysis in either formulations when tested in mice RBCs. These findings confirmed the systemic safety of both PL-NANO and AL-NANO formulations for potential intravenous administration to identify their synergism in BRAFi-resistant melanoma xenograft model in future studies. Also, the stability of both the lipid nanoformulations was tested at 4°C to find no significant difference in their physicochemical properties and drug/nucleic acid encapsulation efficiency rendering them stable for up to 3 months.

Overall, combinatorial delivery of PL-NANO and AL-NANO showed encouraging tumor growth, migration and VM inhibition in 2D cell culture models and displayed substantial reduction in growth of 3D tumor spheroids of acquired as well as intrinsically BRAFi-resistant melanoma cells, suggesting the importance of this therapeutic strategy. Our results indicated strong synergism between both the lipid-based nanocarriers, while substantially improving the therapeutic potency of ARV in AL-NANO to achieve an IC_{50} of as low as 20 nM in A375V cells and 8 nM in RPMI-7951 cells when tested *in vitro*. Importantly, we were able to target critical cellular signaling pathways by significantly upregulating levels of PTEN expression while decreasing the levels of BRD4 and pMAPK/MAPK expression to ultimately gain inhibition of oncogenic c-Myc expression following combinatorial therapy of PL-NANO and AL-NANO in BRAFi-resistant melanoma cells. Both the developed lipid nanoformulations were stable and found to be systemically safe for future *in vivo* evaluation of synergism in drug-resistant melanoma xenograft model. To our knowledge, this is the very first study that explores the anticancer potential of BRD4 PROTAC and PTEN plasmid loaded lipid nanocarriers based combinatorial approach to target c-Myc expression inhibition and provides a promising therapeutic option for drug-resistant melanoma, for which there are no effective treatments at present.

MATERIALS AND METHODS

Materials

ARV-825 (ARV) was purchased from MuseChem (Fairfield, NJ, USA) and VEM was bought from LC Laboratories (Woburn, MA, USA). We procured 1,2-dioleoyl-sn-glycero-3 phosphocholine (DOPC), 1,2-di-(9Z-octadecenoyl)-sn-glycero-3-phosphoethanolamine (DOPE), and 1,2-distearoyl-sn-glycero-3-phosphoethanolamine-N-[amino (polyethylene glycol)-2000]) (DSPE-PEG2000) from Avanti (Alabaster, AL, USA). SM-102 was procured from BroadPharm (San Diego, CA, USA). SDS, dimethyl sulfoxide (DMSO), cholesterol, ultrapure agarose, and crystal violet were purchased from Sigma-Aldrich (St Louis, MO, USA). MTT, DMEM, opti-MEM, citric acid, PBS, and Hank's balanced salt solution were obtained from Thermo Fisher Scientific (Hampton, NH, USA). Fetal bovine serum (FBS) was procured from Atlantic Biologics (Oakwood, GA, USA). Plasmid pCMV Flag WT-PTEN (PTEN-pDNA) was procured from Addgene (Watertown, MA, USA). Plasmid was amplified in *Escherichia coli* (DH5α strain) and purified using PureLink HiPure Plasmid Maxiprep kit (Invitrogen, Waltham, MA), according to the manufacturer's protocol. The concentration and purity of DNA were determined using NanoDrop Onec (Thermo Fisher Scientific).

Development of VEM-resistant melanoma cell lines

Melanoma cell lines (A375 and SK-MEL-28) were purchased from the American Type Culture Collection (Manassas, VA, USA) and were cultured in high glucose DMEM, supplemented with 10% FBS and antibiotic-antimycotic mixture at 37°C with 5% CO_2 in humidified air. VEM-resistant melanoma cell lines (A375V and SK-MEL-28V) were developed by adding VEM (0.2 μM) for up to 20 passages to develop BRAF-mutated VEM-resistant cell lines. Resistance against VEM was confirmed by MTT assay before any *in vitro* studies. The inherently resistant cell line to VEM, RPMI-7951, was obtained from the ATCC and was cultured in high-glucose Eagle's minimum

essential medium , supplemented with 10% FBS and antibiotic-antimycotic mixture at 37°C with 5% CO_2 in humidified air. Developed melanoma cell lines exhibiting intrinsic and acquired resistance were then used for further assays.

HPLC method development for ARV-825

The chromatographic detection of ARV was analyzed using RP-HPLC method as described in the supplemental information (Figure S2).

Identifying synergism between ARV and PTEN plasmid

Synergistic anticancer activity of ARV and PTEN plasmid (PTEN-pDNA) was identified using a commercial transfection reagent Transit LT1, by performing MTT assay in both acquired (A375V) and intrinsically (RPMI-7951) VEM-resistant melanoma cell lines. Briefly, A375V and RPMI-7951 cells were seeded at a density of 5×10^3 cells/well in 96-well plates and were allowed to attach overnight. As a proof-of-concept study, PTEN-pDNA was delivered by using a commercial transfection reagent Transit LT1 at 1:3 ratio of PTEN-pDNA:Transit LT1 (3 μL LT1 per 1 μg PTEN-pDNA). Briefly, cells were transfected with 250 ng PTEN-pDNA per well for 4 h in opti-MEM medium at 37°C. Following transfection, treatment was replaced with varying concentrations of ARV (DMSO stock) containing DMEM medium for 48 h. Similar treatment regimen was followed for ARV without prior transfection with PTEN-pDNA in a separate well plate. After 48 h, cells treated with ARV and PTEN-pDNA alone and in combination were incubated with the MTT solution (5 mg/mL) for 3 h. Following this, the medium was aspirated, and DMSO was added to dissolve the formed MTT-formazan crystals. The MTT-formazan crystals were then quantified by measuring the absorbance at 570 nm on an Epoch2 microplate. IC_{50} values were calculated for both the drugs using GraphPad Prism7 Software.

Fabrication and characterization of ARV-loaded liposomes (AL-NANOs)

Fabrication of AL-NANOs

To prepare AL-NANOs, modified hydration method was used as described in Figure S3, as per the established protocol.[11,18] Briefly, ARV was added to lipid mixture comprising of DOPC: cholesterol:DSPE-PEG 2000 at a molar ratio of 51.1:16.2:1 and dissolved in chloroform to attain a concentration of 1 mg/mL in final liposomal formulation. The lipid and ARV solution was added dropwise to parenteral-grade mannitol (200 μm) (maintained at 50°C) with constant stirring followed by evaporation of organic solvent. Resultant proliposomes powder was dispersed in water with 0.25% w/v citric acid at 55°C followed by probe sonication (30% amplitude) for 2 min to generate unilamellar ARV-loaded liposomes (AL-NANOs). The average particle size, polydispersity index, and zeta potential of AL-NANO were measured via a DLS particle size analyzer (Malvern Zetasizer Nano ZS, Royston, UK) using folded capillary cells. The encapsulation efficiency of AL-NANO was determined by employing the following formula:

$$\%\ \text{Encapsulation efficiency} = \frac{\text{Amount of ARV in AL} - \text{NANO}}{\text{Amount of ARV added}} \times 100\%. \quad \text{(Equation 1)}$$

Cryo-TEM analysis of AL-NANO

The AL-NANO liposomal structure was analyzed using cryo-TEM. Briefly, AL-NANO solution was applied to a copper grid and blotted with a filter paper so a thin liquid film was formed. Blotted samples were instantly plunged into liquid ethane at just above its freezing point (−183°C) using either Leica EM GP cryo-preparation station or Vitrobot. The vitrified samples were then transferred to a TEM using a Gatan (Pleasanton, CA, USA) workstation and cryo-holder for imaging at approximately −183°C. The microscope was operated at 120 kV in a low electron dose mode (to decrease radiation damage) and the images were recorded on a slow scan-cooled charge-coupled device (CCD) camera (Gatan) supported by the Digital Micrograph software package. Images were acquired at multiple scales for each grid to evaluate the overall distribution of AL-NANOs.

In vitro *cytotoxicity of AL-NANOs*

AL-NANOs and ARV in solution were evaluated for their *in vitro* cytotoxicity in A375V (acquired resistance) and RPMI-7951 (intrinsic resistance) cells. Briefly, 1.5×10^4 cells (A375V and RPMI-7951) were seeded per well in 24-well plates and were allowed to attach overnight. After incubation, cells were exposed to various concentrations of ARV in AL-NANO and DMSO stock for a period of 48 h. After 48 h, cell viability was analyzed by performing MTT colorimetric assay, and the IC_{50} values were calculated for AL-NANO and ARV solution using GraphPad Prism7 Software.

Fabrication and characterization of PL-NANOs

Fabrication of PL-NANOs

PL-NANOs were prepared by employing a staggered herringbone micromixer assembly at optimized processing conditions as explained in the supplemental information (Figure S4). Ionizable lipid SM-102, DOPC, DOPE, cholesterol, and DSPE-PEG2000 in a molar ratio of 40:28:10:20.5:1.5 was dissolved in ethanol. For the encapsulation of PTEN-pDNA, the desired amount of PTEN-pDNA was dissolved acetate buffer at pH 4.0 to obtain N/P weight ratio of 60 resulting in PTEN-pDNA concentration of 50 μg/mL. Lipids dissolved in ethanol and PTEN-pDNA solution in buffer were combined in the microfluidic micromixer by means of a dual-syringe pump (model Fusion 100, Chemyx Inc., Stafford, TX, USA). This rapid mixing of both the phases resulted in formation of PL-NANOs, which were dialyzed against 1× PBS, pH 7.4 to remove ethanol using Spectro/Por dialysis membranes (molecular weight cutoff 6–8 kDa, Spectrum Laboratories, Rancho Dominguez, CA, USA). After dialysis, the average particle size, size distribution, and zeta potential of PL-NANO were measured via DLS particle size analyzer (Malvern Zetasizer Nano ZS, Royston, UK) using folded capillary cells.

The PTEN-pDNA entrapment efficiency in PL-NANO was evaluated using the Quant-iT PicoGreen dsDNA Reagent and Kit (Thermo Fisher Scientific). Briefly, equal volume of diluted fluorescent dye was added to the diluted PL-NANO in the presence or absence of 1% (w/v) Triton X-100 in TE buffer following incubation for 5 min. Fluorescence units of lysed PL-NANO was fit to a known standard curve of PTEN-pDNA established using the same kit. Entrapment of PTEN-pDNA in PL-NANO was quantified by comparing the fluorescence value of PL-NANO (P_i) to that of total PTEN-pDNA content obtained upon lysis of PL-NANO by Triton X-100 (P_t) using the following formula:

$$\%\ \text{Entrapment efficiency} = \frac{P_t - P_i}{P_t} \times 100\%. \qquad \text{(Equation 2)}$$

The PL-NANO formulation was then passed through a 0.22-μm membrane filter to perform further *in vitro* assays and stored at 4°C until use.

Electrophoretic mobility shift assay

To evaluate the PTEN-pDNA binding affinity toward PL-NANO, agarose gel electrophoresis was performed. Briefly, PL-NANO and PTEN-pDNA extracted from PL-NANO were added into 6× loading buffer and loaded onto a 1% (w/v) agarose gel containing SYBR green dye. Electrophoresis was performed at 100 V for 1 h in TAE buffer, and the PTEN-pDNA bands were viewed under ultraviolet illumination using Azure biosystems c500 imager. Naked PTEN-pDNA was used as a control.

Cryo-TEM analysis of PL-NANO

The structural analysis of PL-NANO was performed via Cryo-TEM. Briefly, PL-NANO solution was applied to a copper grid and blotted with a filter paper to form a thin liquid film. Blotted samples were vitrified and examined at 120 kV in a low electron dose mode (to reduce radiation damage) and the images were recorded on a slow scan cooled CCD camera (Gatan) supported by the Digital Micrograph software package. Images were acquired at multiple scales for each grid to evaluate the overall distribution of PL-NANOs.

Cell counting assay of PL-NANOs

The cell counting assay was performed to determine tumor growth inhibition by PL-NANO in A375V cells. Briefly, A375V cells were seeded at a density of 1.5×10^4 cells/well in 24-well plates and were allowed to attach overnight. Following incubation, cells were transfected with PL-NANO at 0.5 and 1 μg PTEN-pDNA per well in opti-MEM for 4 h at 37°C. After 4 h, treatment was replaced with fresh DMEM media and cells were counted every 24 h for 3 days. Trypan blue exclusion method was used to determine the number of live cells and effect of PL-NANO on growth of A375V cells was observed in comparison with control or untreated cells.

ELISA for PTEN expression by PL-NANO

Target protein expression mediated by PL-NANO was analyzed using PTEN Colorimetric Cell-Based ELISA Kit (Boster Bio, Pleasanton, CA, USA). This assay is based on an indirect ELISA format to determine the target PTEN protein. Given the reported downregulation or loss of PTEN tumor suppressor gene in melanoma cells with acquired resistance against BRAFi, A375V cells were used to determine the PTEN gene delivery efficiency of PL-NANO by following the manufacturer's protocol. A375V cells were seeded at a density of 5×10^3 cells/well in a 96-well plate and incubated overnight. Cells were transfected with PL-NANO at 100 ng and 250 ng of PTEN-pDNA per well for 4 h in opti-MEM medium at 37°C. Commercial transfection reagent Transit LT1 was used as a positive control at 1:3 ratio of PTEN-pDNA:Transit LT1 to attain target protein expression. After 4 h, treatment was replaced with fresh DMEM medium for 48 h. At 48 h of treatment, ELISA was performed as per the manufacturer's protocol. Briefly, treatment was removed and cells were washed with 1× Tris-buffered saline followed by fixation with 4% v/v glutaraldehyde. After fixation, quenching buffer was added, cells were washed with wash buffer and incubated with the blocking buffer for 1 h at room temperature (RT). Following this, 1× primary anti-PTEN antibody was added to treated wells, while anti-GAPDH antibody was added to control/untreated wells and incubated overnight at 4°C. Then, cells were washed with wash buffer and incubated with 1× secondary horseradish peroxidase (HRP)-conjugated anti-rabbit IgG antibody for treated wells, and HRP-conjugated anti-mouse IgG antibody for control/untreated wells for 1.5 h at RT with gentle shaking on the shaker. Finally, substrate was added to each well for 30 min at RT, which resulted in a colorimetric reaction. Following incubation, stop solution was added to each well and absorbance was read at 450 nm immediately using a UV spectrophotometer.

Synergism between AL-NANO and PL-NANO in BRAFi-resistant melanoma cell lines

Cell viability assay

The cytotoxicity of AL-NANO and PL-NANO were evaluated in A375V and RPMI-7951 cells. A375V and RPMI-7951 cells were seeded at a density of 1.5×10^4 cells per well in 24-well plates and incubated overnight at 37°C and 5% CO_2. Following incubation, cells were transfected with PL-NANO at 0.5 and 1 μg PTEN-pDNA per well in opti-MEM for 4 h at 37°C. After 4 h, PL-NANO treatment was replaced with various concentrations of ARV in AL-NANO and incubated for another 48 h. Similar treatment regimen was followed for AL-NANO without prior transfection with PL-NANO in a separate well-plate. After 48 h of treatment, cell viability was assessed using MTT assay and IC_{50} of ARV in AL-NANO was calculated alone and that following PL-NANO treatment in both the cell lines using GraphPad Prism7 Software.

In vitro *migration assay*

In vitro migration assay was performed in A375V cells. For this, cells were seeded at a density of 1.5×10^3 cells per well in a 96-well plate and incubated overnight. Once the cells were confluent, a uniform scratch was made in each well with a sterile 200-μL pipette tip. Cells were treated with PL-NANO (0.25 μg PTEN-pDNA), AL-NANO (10 nM ARV), and their combination for 24 h. Briefly, cells were initially transfected with PL-NANO for 4 h in opti-MEM. After 4

h, PL-NANO treatment was replaced with fresh DMEM media (alone group) or AL-NANO containing DMEM media (combination group) and incubated for another 24 h. Following 24 h of incubation, treatment was removed, and cells were washed with PBS. Then, cells were fixed in 4% v/v glutaraldehyde followed by staining with 0.5% w/v crystal violet dye for 30 min at RT. Cells were thoroughly washed with sterile water and plates were air-dried overnight. Images of scratch area were captured and percent bridging of migration area for PL-NANO, AL-NANO, and combination treated cells was determined using ImageJ software.

Clonogenic survival assay

Clonogenic assay was carried out in A375V cells following treatment with PL-NANO and AL-NANO. Initially, cells were seeded at a density of 1,000 cells per well in a 6-well plate and incubated for 24 h at 37°C. Then, cells were treated with PL-NANO (0.25 μg PTEN-pDNA), AL-NANO (10 nM ARV), and their combination for 24 h. Briefly, cells were transfected with PL-NANO for 4 h in opti-MEM. After 4 h, PL-NANO treatment was replaced with fresh DMEM media (alone group) or AL-NANO containing DMEM media (combination group) and incubated for another 24 h. Following 24 h, the treatment was replaced with fresh DMEM media, and the cells were allowed to grow until sufficiently large colonies were formed in the control wells. Once the colonies were formed, cells were fixed with 4% v/v glutaraldehyde followed by their staining with 0.5% w/v crystal violet for 30 min at RT. Cells were then washed thoroughly with sterile water and plates were air-dried overnight. The number of colonies present in the treatment and control groups were counted manually by the colony counting method.

VM assay

Since A375V cells have the tendency to exhibit vasculogenic mimicry, this cell line was to identify the inhibitory effect of PL-NANO and AL-NANO on *in vitro* VM. Initially, a 96-well plate was coated with 50 μL Basement Membrane Extract (BME). Then, suspension of A375V cells at 2 × 10^5/mL was incubated with different treatment groups including PL-NANO (0.25 μg PTEN-pDNA), AL-NANO (25 nM ARV), and their combination for 5 min at RT. Cell suspension in presence and absence of various treatments was in the pre-coated 96-well plate at a density of 2 × 10^4 cells/well. After 24 h of incubation at 37°C with 5% CO_2, bright field images were taken using an EVOS light microscope and number of branching points present in each group were quantified to denote tube formation.

Flow cytometry for in vitro *apoptosis analysis*

In vitro apoptosis was analyzed in A375V and RPMI-7951 cells following treatment with PL-NANO and AL-NANO. Cells were seeded at a density of 8 × 10^4/mL in 12-well plate and incubated overnight. Cells were treated with PL-NANO (0.5 μg PTEN-pDNA), AL-NANO (50 nM ARV), and their combination for 48 h. As mentioned earlier, cells were initially transfected with PL-NANO for 4 h in opti-MEM followed by replacement with DMEM media or treatment with AL-NANO for alone and combination groups, respectively. After 48 h of incubation, cells were trypsinized, centrifuged and resuspended in DMEM media (containing 1% bovine serum albumin and 1% FBS) to a concentration of 1 × 10^6 cells/mL. Apoptosis analysis was performed by using Muse Annexin V & Dead Cell Assay kit (MilliporeSigma, Billerica, MA, USA). Before analysis, the cell suspension was diluted twice with MUSE Annexin V and dead cell reagent, and incubated for 20 min at RT. Following incubation, the samples were analyzed for apoptosis using Muse Cell Analyzer (MilliporeSigma).

Development and cell viability of 3D MCTSs after treatment with AL-NANO and PL-NANO

The 3D MCTS were developed for both A375V and RPMI-7951 cell lines by seeding cells at a density of 1.5 x 10^3 cells/well in an ultra-low attachment 96-well plate (Corning Life Sciences, Bedford, MA, USA) followed by centrifugation at 140×*g* for 10 min[51,52] Spheroids were allowed to form by culturing in complete DMEM media at 37°C for 72 h. Following this, spheroids were treated with PL-NANO (2 μg PTEN-pDNA), AL-NANO (200 nM ARV), and their combination. Cells were initially transfected with PL-NANO for 4 h in opti-MEM followed by replacement with DMEM media or treatment with AL-NANO for alone and combination groups, respectively. Fresh treatment was replaced every alternate day for up to 5 days. During the treatment period, spheroids were analyzed for their growth and morphology in each treatment group as compared with control. On the fifth day of treatment, spheroids were stained with a mixture of three dyes: 1 μM calcein AM, 3 μM EthD-1 and 4 μM DAPI using a Viability/Cytotoxicity Assay Kit (Biotium) and incubated for 3 h at 37°C before imaging. Fluorescent images were captured for each group using an Evos fluorescence microscope (Thermo Fisher Scientific, Waltham, MA, USA).

Western blot assay

A375V cells were analyzed for target protein expression following treatment with PL-NANO and AL-NANO. Briefly, cells were treated with PL-NANO (10 μg PTEN-pDNA) and AL-NANO (200 nM ARV), alone and in combination, for 48 h. PL-NANO was initially transfected in cells for 4 h in opti-MEM followed by addition of AL-NANO. Following treatment, cells were lysed in modified RIPA buffer (50 mM Tris pH 8.0, 150 mM NaCl, 1% v/v NP40, 0.5% w/v deoxycholate, 0.1% w/v SDS, 10% v/v glycerol, 10 mM NaF, 0.4 mM EDTA) with protease inhibitors, followed by centrifugation at 4°C for 10 min at 10,000×*g*. The supernatant was collected and Laemmli sample buffer containing SDS and β-mercaptoethanol was added. Samples were denatured by heating at 95°C for 10 min. Consequently, samples were separated on polyacrylamide gels and transferred to PVDF membrane, and probed with primary antibodies BRD4 (13440), c-Myc (5605), PTEN (9559), *p*-MAPK (4370), and MAPK (9102) from Cell Signaling Technology, and β-actin antibody (66009) from Proteintech (Rosemont, IL, USA). HRP-conjugated secondary antibodies were used along with enhanced chemiluminescence substrate. Images were obtained with the Azure C500 imaging system and quantified using ImageJ 1.8.0 software.

Biocompatibility and hemocompatibility of AL-NANO and PL-NANO

To analyze the biocompatibility of both AL-NANO and PL-NANO, their toxic effects were analyzed in non-cancerous MDCK cells. Briefly, MDCK cells were seeded at a density of 1.5×10^4 cells per well in a 96-well plate to form a monolayer and incubated overnight at 37°C and 5% CO_2. Following incubation, cells were transfected with PL-NANO at 0.5 μg PTEN-pDNA per well in opti-MEM for 4 h at 37°C. After 4 h, PL-NANO treatment was replaced with various concentrations of ARV in AL-NANO and incubated for another 24 h. Similar treatment regimen was followed for AL-NANO without prior transfection with PL-NANO in a separate well-plate. After 24 h of treatment, cell viability was assessed using an MTT assay.

Mice RBCs were used to test the *in vitro* hemolysis of AL-NANO and PL-NANO. C57BL/6 mice (5–6 weeks old) from Jackson Laboratories (Bar Harbor, ME, USA) were used for the experimental protocol that was approved by the St. John's University Institutional Animal Care and Use Committee. Briefly, mice were euthanized by carbon dioxide followed by a one-time blood collection using the cardiac puncture technique. RBCs were separated by centrifugation was performed at 450×g for 10 min and resultant cell pellet was washed with and redispersed into PBS to achieve the same hematocrit. PL-NANO at SM-102 ionizable lipid concentration of 200 μM and AL-NANO at 10 μg/mL ARV concentration were added to the RBC dispersion followed by incubation for 30 min at 37°C. Centrifugation was performed at 450×*g* for 10 min and supernatant was diluted with PBS for analysis of hemoglobin release using a UV spectrophotometer at 550 nm. Controls used for this experiment were PBS (negative control) and sodium lauryl sulfate solution (positive control). Percentage hemolysis exhibited by PL-NANO and AL-NANO was determined by the following formula:

$$\begin{aligned}\%\ \text{Hemolysis} = & \ (\text{Absorbance of test sample} \\ & - \text{Absorbance of negative control}) \\ & \div (\text{Absorbance of positive control} \\ & - \text{Absorbance of negative control}) \times 100\%.\end{aligned} \quad \text{(Equation 3)}$$

Stability analysis of AL-NANO and PL-NANO

Freshly prepared AL-NANO and PL-NANO were investigated for their physical stability in terms of particle size, zeta-potential and encapsulation efficiency (%) at different time points for a period of 3 months. During this period of 3 months, both the lipid nanoformulations were stored at 4°C.

Statistical analyses

Each experiment is performed in triplicate and all data shown are reported as the mean ± SD. One-way ANOVA followed by Bonferroni's or Dunnett's multiple comparison tests were used to perform all statistical analyses using GraphPad Prism7 (GraphPad Software, La Jolla, CA, USA). Statistically significant difference between various groups is indicated by a p value of less than 0.05.

DATA AND CODE AVAILABILITY

Data will be made available on request.

SUPPLEMENTAL INFORMATION

Supplemental information can be found online at https://doi.org/10.1016/j.omtn.2024.102292.

ACKNOWLEDGMENTS

This work was supported by College of Pharmacy and Health Sciences, St. John's University, Queens, NY, USA.
This work was partially funded by the National Institutes of Health (NIH)- NIGMS grants SC2 [1SC2GM130478] and R16 [1R16GM145556-01A1].

AUTHOR CONTRIBUTIONS

A.S.: Data curation, Formal analysis, Investigation, Methodology, Software, Writing - original draft; H.P.V.: Methodology; V.D.: Supervision; Validation; K.P.: Conceptualization; Funding acquisition; Investigation; Project administration; Resources; Software; Supervision; Validation; Visualization; Writing - review & editing.

DECLARATION OF INTERESTS

The authors declare no competing interests.

REFERENCES

1. Siegel, R.L., Miller, K.D., Wagle, N.S., and Jemal, A. (2023). Cancer statistics, 2023. CA. Cancer J. Clin. *73*, 17–48. https://doi.org/10.3322/caac.21763.
2. Domingues, B., Lopes, J.M., Soares, P., and Pópulo, H. (2018). Melanoma treatment in review. ImmunoTargets Ther. *7*, 35–49. https://doi.org/10.2147/ITT.S134842.
3. Kozar, I., Margue, C., Rothengatter, S., Haan, C., and Kreis, S. (2019). Many ways to resistance: How melanoma cells evade targeted therapies. Biochim. Biophys. Acta. Rev. Cancer *1871*, 313–322. https://doi.org/10.1016/j.bbcan.2019.02.002.
4. Patel, M., Eckburg, A., Gantiwala, S., Hart, Z., Dein, J., Lam, K., and Puri, N. (2021). Resistance to Molecularly Targeted Therapies in Melanoma. Cancers *13*, 1115. https://doi.org/10.3390/cancers13051115.
5. Singleton, K.R., Crawford, L., Tsui, E., Manchester, H.E., Maertens, O., Liu, X., Liberti, M.V., Magpusao, A.N., Stein, E.M., Tingley, J.P., et al. (2017). Melanoma Therapeutic Strategies that Select against Resistance by Exploiting MYC-Driven Evolutionary Convergence. Cell Rep. *21*, 2796–2812. https://doi.org/10.1016/j.celrep.2017.11.022.
6. Liu, Z., Hu, M., Yang, Y., Du, C., Zhou, H., Liu, C., Chen, Y., Fan, L., Ma, H., Gong, Y., and Xie, Y. (2022). An overview of PROTACs: a promising drug discovery paradigm. Mol. Biomed. *3*, 46. https://doi.org/10.1186/s43556-022-00112-0.
7. Saraswat, A.L., Vartak, R., Hegazy, R., Patel, A., and Patel, K. (2023). Drug delivery challenges and formulation aspects of proteolysis targeting chimera (PROTACs). Drug Discov. Today *28*, 103387. https://doi.org/10.1016/j.drudis.2022.103387.
8. Sincere, N.I., Anand, K., Ashique, S., Yang, J., and You, C. (2023). PROTACs: Emerging Targeted Protein Degradation Approaches for Advanced Druggable Strategies. Molecules *28*, 4014. https://doi.org/10.3390/molecules28104014.
9. Ambrosini, G., Sawle, A.D., Musi, E., and Schwartz, G.K. (2015). BRD4-targeted therapy induces Myc-independent cytotoxicity in Gnaq/11-mutatant uveal melanoma cells. Oncotarget *6*, 33397–33409. https://doi.org/10.18632/oncotarget.5179.
10. Kotekar, A., Singh, A.K., and Devaiah, B.N. (2023). BRD4 and MYC: power couple in transcription and disease. FEBS J. *290*, 4820–4842. https://doi.org/10.1111/febs.16580.
11. Fu, Y., Saraswat, A., Wei, Z., Agrawal, M.Y., Dukhande, V.V., Reznik, S.E., and Patel, K. (2021). Development of Dual ARV-825 and Nintedanib-Loaded PEGylated

Nano-Liposomes for Synergistic Efficacy in Vemurafnib-Resistant Melanoma. Pharmaceutics *13*, 1005. https://doi.org/10.3390/pharmaceutics13071005.

12. Rathod, D., Fu, Y., and Patel, K. (2019). BRD4 PROTAC as a novel therapeutic approach for the treatment of vemurafenib resistant melanoma: Preformulation studies, formulation development and in vitro evaluation. Eur. J. Pharm. Sci. *138*, 105039. https://doi.org/10.1016/j.ejps.2019.105039.
13. Zuo, Q., Liu, J., Huang, L., Qin, Y., Hawley, T., Seo, C., Merlino, G., and Yu, Y. (2018). AXL/AKT axis mediated-resistance to BRAF inhibitor depends on PTEN status in melanoma. Oncogene *37*, 3275–3289. https://doi.org/10.1038/s41388-018-0205-4.
14. Rascio, F., Spadaccino, F., Rocchetti, M.T., Castellano, G., Stallone, G., Netti, G.S., and Ranieri, E. (2021). The Pathogenic Role of PI3K/AKT Pathway in Cancer Onset and Drug Resistance: An Updated Review. Cancers *13*, 3949. https://doi.org/10.3390/cancers13163949.
15. Vidarsdottir, L., Azimi, A., Das, I., Sigvaldadottir, I., Suryo Rahmanto, A., Petri, A., Kauppinen, S., Ingvar, C., Jönsson, G., Olsson, H., et al. (2021). PTENP1-AS contributes to BRAF inhibitor resistance and is associated with adverse clinical outcome in stage III melanoma. Sci. Rep. *11*, 11023. https://doi.org/10.1038/s41598-021-89389-9.
16. Price, E., Weinheimer, M., Rivkin, A., Jenkins, G., Nijsen, M., Cox, P.B., and DeGoey, D. (2024). Beyond Rule of Five and PROTACs in Modern Drug Discovery: Polarity Reducers, Chameleonicity, and the Evolving Physicochemical Landscape. J. Med. Chem. *67*, 5683–5698. https://doi.org/10.1021/acs.jmedchem.3c02332.
17. Saraswat, A., Patki, M., Fu, Y., Barot, S., Dukhande, V.V., and Patel, K. (2020). Nanoformulation of PROteolysis TArgeting Chimera targeting 'undruggable' c-Myc for the treatment of pancreatic cancer. Nanomedicine (Lond) *15*, 1761–1777. https://doi.org/10.2217/nnm-2020-0156.
18. Saraswat, A., Vemana, H.P., Dukhande, V.V., and Patel, K. (2022). Galactose-decorated liver tumor-specific nanoliposomes incorporating selective BRD4-targeted PROTAC for hepatocellular carcinoma therapy. Heliyon *8*, e08702. https://doi.org/10.1016/j.heliyon.2021.e08702.
19. Kou, P., Levy, E.S., Nguyen, A.D., Zhang, D., Chen, S., Cui, Y., Zhang, X., Broccatelli, F., Pizzano, J., Cantley, J., et al. (2023). Development of Liposome Systems for Enhancing the PK Properties of Bivalent PROTACs. Pharmaceutics *15*, 2098. https://doi.org/10.3390/pharmaceutics15082098.
20. Liu, P., Chen, G., and Zhang, J. (2022). A Review of Liposomes as a Drug Delivery System: Current Status of Approved Products, Regulatory Environments, and Future Perspectives. Molecules *27*, 1372. https://doi.org/10.3390/molecules27041372.
21. Pozzi, D., and Caracciolo, G. (2023). Looking Back, Moving Forward: Lipid Nanoparticles as a Promising Frontier in Gene Delivery. ACS Pharmacol. Transl. Sci. *6*, 1561–1573. https://doi.org/10.1021/acsptsci.3c00185.
22. Yonezawa, S., Koide, H., and Asai, T. (2020). Recent advances in siRNA delivery mediated by lipid-based nanoparticles. Adv. Drug Deliv. Rev. *154–155*, 64–78. https://doi.org/10.1016/j.addr.2020.07.022.
23. Suzuki, Y., and Ishihara, H. (2021). Difference in the lipid nanoparticle technology employed in three approved siRNA (Patisiran) and mRNA (COVID-19 vaccine) drugs. Drug Metab. Pharmacokinet. *41*, 100424. https://doi.org/10.1016/j.dmpk.2021.100424.
24. Ansari, M.T., Ramlan, T.A., Jamaluddin, N.N., Zamri, N., Salfi, R., Khan, A., Sami, F., Majeed, S., and Hasnain, M.S. (2020). Lipid-based Nanocarriers for Cancer and Tumor Treatment. Curr. Pharm. Des. *26*, 4272–4276. https://doi.org/10.2174/1381612826666200720235752.
25. Saraswat, A., and Patel, K. (2023). Delineating effect of headgroup and preparation method on transfection versus toxicity of DNA-loaded lipid nanocarriers. Nanomedicine (Lond) *18*, 1921–1940. https://doi.org/10.2217/nnm-2023-0219.
26. Llombart, V., and Mansour, M.R. (2022). Therapeutic targeting of "undruggable" MYC. EBioMedicine *75*, 103756. https://doi.org/10.1016/j.ebiom.2021.103756.
27. Han, H., Jain, A.D., Truica, M.I., Izquierdo-Ferrer, J., Anker, J.F., Lysy, B., Sagar, V., Luan, Y., Chalmers, Z.R., Unno, K., et al. (2019). Small-Molecule MYC Inhibitors Suppress Tumor Growth and Enhance Immunotherapy. Cancer Cell *36*, 483–497.e15. https://doi.org/10.1016/j.ccell.2019.10.001.
28. Li, X., Zhang, Z., Gao, F., Ma, Y., Wei, D., Lu, Z., Chen, S., Wang, M., Wang, Y., Xu, K., et al. (2023). c-Myc-Targeting PROTAC Based on a TNA-DNA Bivalent Binder for Combination Therapy of Triple-Negative Breast Cancer. J. Am. Chem. Soc. *145*, 9334–9342. https://doi.org/10.1021/jacs.3c02619.
29. Pastorino, F., Brignole, C., Marimpietri, D., Pagnan, G., Morando, A., Ribatti, D., Semple, S.C., Gambini, C., Allen, T.M., and Ponzoni, M. (2003). Targeted liposomal c-myc antisense oligodeoxynucleotides induce apoptosis and inhibit tumor growth and metastases in human melanoma models. Clin. Cancer Res. *9*, 4595–4605.
30. Yao, R., Zhang, M., Zhou, J., Liu, L., Zhang, Y., Gao, J., and Xu, K. (2022). Novel dual-targeting c-Myc inhibitor D347-2761 represses myeloma growth via blocking c-Myc/Max heterodimerization and disturbing its stability. Cell Commun. Signal. *20*, 73. https://doi.org/10.1186/s12964-022-00868-6.
31. Chen, D., Liu, X., Lu, X., and Tian, J. (2023). Nanoparticle drug delivery systems for synergistic delivery of tumor therapy. Front. Pharmacol. *14*, 1111991. https://doi.org/10.3389/fphar.2023.1111991.
32. Swami, R., Kumar, Y., Chaudhari, D., Katiyar, S.S., Kuche, K., Katare, P.B., Banerjee, S.K., and Jain, S. (2021). pH sensitive liposomes assisted specific and improved breast cancer therapy using co-delivery of SIRT1 shRNA and Docetaxel. Mater. Sci. Eng. C Mater. Biol. Appl. *120*, 111664. https://doi.org/10.1016/j.msec.2020.111664.
33. Younis, M.A., Khalil, I.A., Elewa, Y.H.A., Kon, Y., and Harashima, H. (2021). Ultra-small lipid nanoparticles encapsulating sorafenib and midkine-siRNA selectively-eradicate sorafenib-resistant hepatocellular carcinoma in vivo. J. Control. Release *331*, 335–349. https://doi.org/10.1016/j.jconrel.2021.01.021.
34. Eygeris, Y., Patel, S., Jozic, A., and Sahay, G. (2020). Deconvoluting Lipid Nanoparticle Structure for Messenger RNA Delivery. Nano Lett. *20*, 4543–4549. https://doi.org/10.1021/acs.nanolett.0c01386.
35. Li, S., Hu, Y., Li, A., Lin, J., Hsieh, K., Schneiderman, Z., Zhang, P., Zhu, Y., Qiu, C., Kokkoli, E., et al. (2022). Payload distribution and capacity of mRNA lipid nanoparticles. Nat. Commun. *13*, 5561. https://doi.org/10.1038/s41467-022-33157-4.
36. Patel, K., Doddapaneni, R., Chowdhury, N., Boakye, C.H., Behl, G., and Singh, M. (2016). Tumor stromal disrupting agent enhances the anticancer efficacy of docetaxel loaded PEGylated liposomes in lung cancer. Nanomedicine (Lond) *11*, 1377–1392. https://doi.org/10.2217/nnm.16.37.
37. Luo, Q., Wang, J., Zhao, W., Peng, Z., Liu, X., Li, B., Zhang, H., Shan, B., Zhang, C., and Duan, C. (2020). Vasculogenic mimicry in carcinogenesis and clinical applications. J. Hematol. Oncol. *13*, 19. https://doi.org/10.1186/s13045-020-00858-6.
38. Wei, X., Chen, Y., Jiang, X., Peng, M., Liu, Y., Mo, Y., Ren, D., Hua, Y., Yu, B., Zhou, Y., et al. (2021). Mechanisms of vasculogenic mimicry in hypoxic tumor microenvironments. Mol. Cancer *20*, 7. https://doi.org/10.1186/s12943-020-01288-1.
39. Lin, X., Sun, R., Zhao, X., Zhu, D., Zhao, X., Gu, Q., Dong, X., Zhang, D., Zhang, Y., Li, Y., and Sun, B. (2017). C-myc overexpression drives melanoma metastasis by promoting vasculogenic mimicry via c-myc/snail/Bax signaling. J. Mol. Med. *95*, 53–67. https://doi.org/10.1007/s00109-016-1452-x.
40. Han, S.J., Kwon, S., and Kim, K.S. (2021). Challenges of applying multicellular tumor spheroids in preclinical phase. Cancer Cell Int. *21*, 152. https://doi.org/10.1186/s12935-021-01853-8.
41. Nunes, A.S., Barros, A.S., Costa, E.C., Moreira, A.F., and Correia, I.J. (2019). 3D tumor spheroids as in vitro models to mimic in vivo human solid tumors resistance to therapeutic drugs. Biotechnol. Bioeng. *116*, 206–226. https://doi.org/10.1002/bit.26845.
42. Sirenko, O., Mitlo, T., Hesley, J., Luke, S., Owens, W., and Cromwell, E.F. (2015). High-content assays for characterizing the viability and morphology of 3D cancer spheroid cultures. Assay Drug Dev. Technol. *13*, 402–414. https://doi.org/10.1089/adt.2015.655.
43. Nakdimon, I., Walser, M., Fröhli, E., and Hajnal, A. (2012). PTEN negatively regulates MAPK signaling during Caenorhabditis elegans vulval development. PLoS Genet. *8*, e1002881. https://doi.org/10.1371/journal.pgen.1002881.
44. Kotelevets, L., van Hengel, J., Bruyneel, E., Mareel, M., van Roy, F., and Chastre, E. (2001). The lipid phosphatase activity of PTEN is critical for stabilizing intercellular junctions and reverting invasiveness. J. Cell Biol. *155*, 1129–1135. https://doi.org/10.1083/jcb.200105109.
45. Wu, X., Hepner, K., Castelino-Prabhu, S., Do, D., Kaye, M.B., Yuan, X.J., Wood, J., Ross, C., Sawyers, C.L., and Whang, Y.E. (2000). Evidence for regulation of the PTEN tumor suppressor by a membrane-localized multi-PDZ domain containing scaffold protein MAGI-2. Proc. Natl. Acad. Sci. USA *97*, 4233–4238. https://doi.org/10.1073/pnas.97.8.4233.

46. Cecchini, C., Pannilunghi, S., Tardy, S., and Scapozza, L. (2021). From Conception to Development: Investigating PROTACs Features for Improved Cell Permeability and Successful Protein Degradation. Front. Chem. *9*, 672267. https://doi.org/10.3389/fchem.2021.672267.

47. Nishiguchi, G., Keramatnia, F., Min, J., Chang, Y., Jonchere, B., Das, S., Actis, M., Price, J., Chepyala, D., Young, B., et al. (2021). Identification of Potent, Selective, and Orally Bioavailable Small-Molecule GSPT1/2 Degraders from a Focused Library of Cereblon Modulators. J. Med. Chem. *64*, 7296–7311. https://doi.org/10.1021/acs.jmedchem.0c01313.

48. Wang, J., Li, G.L., Ming, S.L., Wang, C.F., Shi, L.J., Su, B.Q., Wu, H.T., Zeng, L., Han, Y.Q., Liu, Z.H., et al. (2020). BRD4 inhibition exerts anti-viral activity through DNA damage-dependent innate immune responses. PLoS Pathog. *16*, e1008429. https://doi.org/10.1371/journal.ppat.1008429.

49. Mukhopadhyay, S., Veroniaina, H., Chimombe, T., Han, L., Zhenghong, W., and Xiaole, Q. (2019). Synthesis and compatibility evaluation of versatile mesoporous silica nanoparticles with red blood cells: an overview. RSC Adv. *9*, 35566–35578. https://doi.org/10.1039/c9ra06127d.

50. Sharma, A., Madhunapantula, S.V., and Robertson, G.P. (2012). Toxicological considerations when creating nanoparticle-based drugs and drug delivery systems. Expert Opin. Drug Metab. Toxicol. *8*, 47–69. https://doi.org/10.1517/17425255.2012.637916.

51. Saraswat, A., and Patel, K. (2024). Development and in-depth characterization of BRAFi-resistant melanoma cell lines in vitro and in vivo. Exp. Cell Res. *438*, 114033. https://doi.org/10.1016/j.yexcr.2024.114033.

52. Saraswat, A., Vartak, R., Hegazy, R., Fu, Y., Rao, T.J.R., Billack, B., and Patel, K. (2023). Oral lipid nanocomplex of BRD4 PROteolysis TArgeting Chimera and vemurafenib for drug-resistant malignant melanoma. Biomed. Pharmacother. *168*, 115754. https://doi.org/10.1016/j.biopha.2023.115754.

OMTN, Volume *35*

Supplemental information

Novel gene therapy for drug-resistant melanoma: Synergistic combination of PTEN plasmid and BRD4 PROTAC-loaded lipid nanocarriers

Aishwarya Saraswat, Hari Priya Vemana, Vikas Dukhande, and Ketan Patel

Methods

HPLC method development for ARV-825

The chromatographic detection of ARV was analyzed using Waters alliance system equipped with 2998 Photodiode Array (PDA) detector and InertSustain C18 column (150 mm × 4.6 mm, 5 µm) (GL Sciences, CA, USA). Acetonitrile and potassium dihydrogen phosphate buffer of pH 3.5 at a volume ratio of 60:40 was used as the mobile phase at a flow rate of 1 mL/min and the injection volume was 10 µL. The output signal was monitored and processed using Empower 3 software. The column temperature was kept at 25°C and ARV was detected at a wavelength of 247 nm. The optimized mobile phase ratio resulted in a sharp peak of ARV with a retention time of 6.7 ± 0.2 min as shown in Fig. S2.

Table S1. *IC_{50} values of ARV alone and in combination with PTEN transfected via Transit LT1 in BRAFi-resistant melanoma cell lines. (n = 6, Data are represented as mean ± SD).*

**Cell line**	**IC_{50} of ARV (μM)**		**Fold decrease in IC_{50} of ARV**
	**ARV alone**	**ARV+PTEN**	
A375V	0.08 ± 0.01	0.01 ± 0.005	~8.0
RPMI-7951	0.50 ± 0.11	0.014 ± 0.003	~15.0

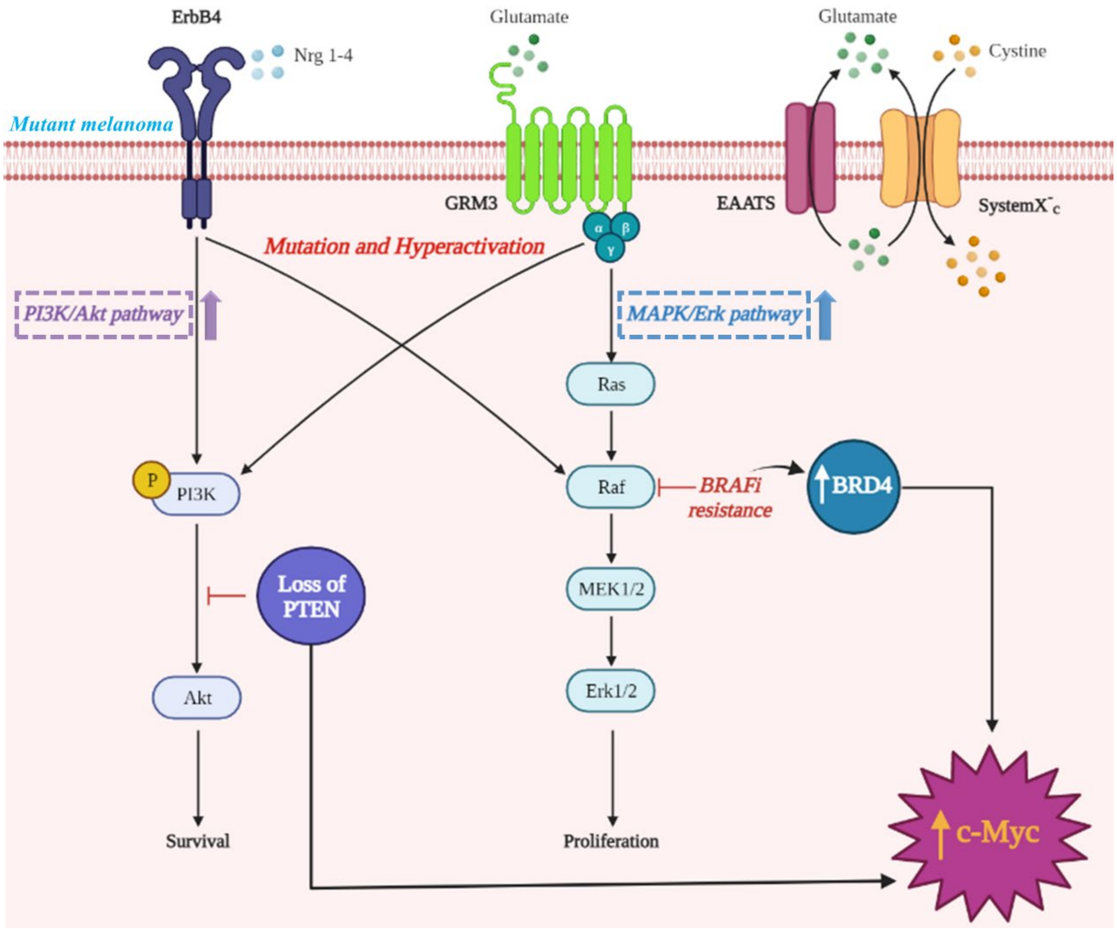

Fig. S1. Targeting of two key signaling pathways responsible for development of resistance against BRAFi in mutant melanoma. *Hyperactivation of major cellular signaling pathways including MAPK/ERK and PI3K/Akt pathways result in upregulation of BRD4 protein and loss of PTEN gene, respectively, to finally converge to c-Myc activation.*

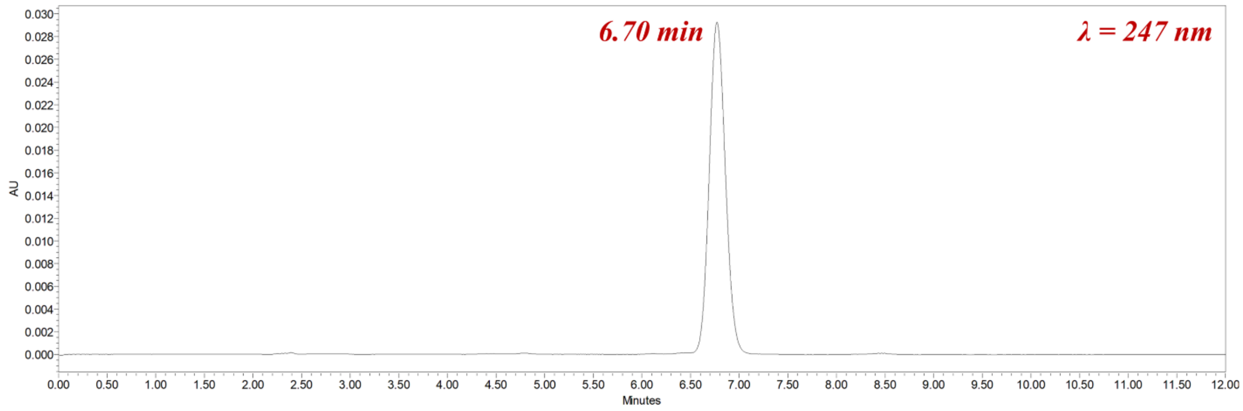

Fig. S2. Chromatogram of ARV corresponding to 10 ppm concentration as analyzed by HPLC. *ARV was found to be retained at 6.70 min to give a sharp peak when detected at a wavelength of 247 nm.*

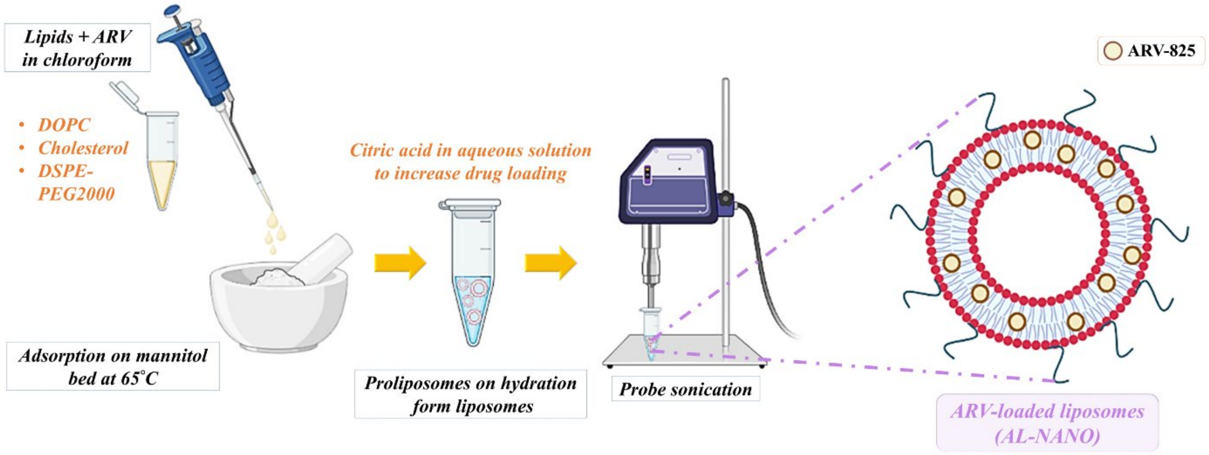

Fig. S3. Schematic representation for preparation of ARV-loaded liposomes (AL-NANO) by modified hydration method. *Lipids and ARV were dissolved in chloroform and adsorbed on mannitol bed. Following evaporation of organic solvent, resultant proliposomes were hydrated with citric acid solution and probe sonicated to form ARV-NANO that were used to perform in vitro assays.*

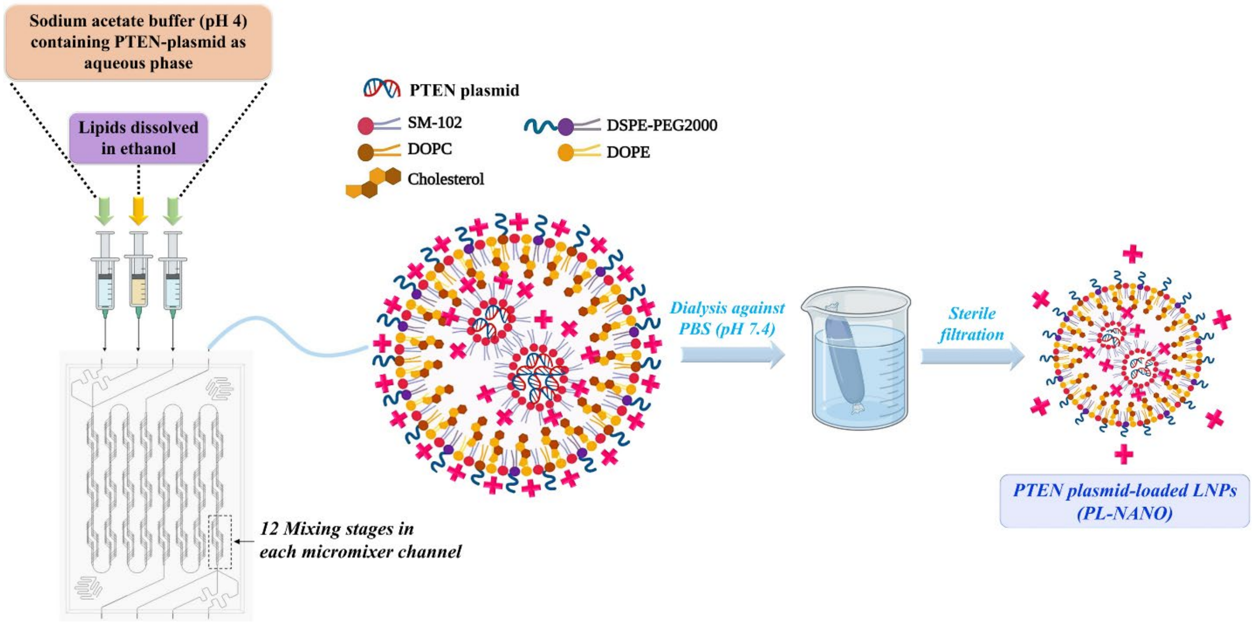

Fig. S4. Schematic representation for preparation of PTEN plasmid-loaded LNPs (PL-NANO) by microfluidic mixing approach. *Lipids dissolved in ethanol and PTEN-pDNA in buffer as aqueous solution are pumped into the two inlets of the microfluidic mixer using a syringe pump. Staggered herringbone structures induce chaotic mixing of both phases and correspondingly rapid increases in the polarity of the lipid solution to form PL-NANO. PL-NANO are further subjected to dialysis and sterile filtration for in vitro experiments.*

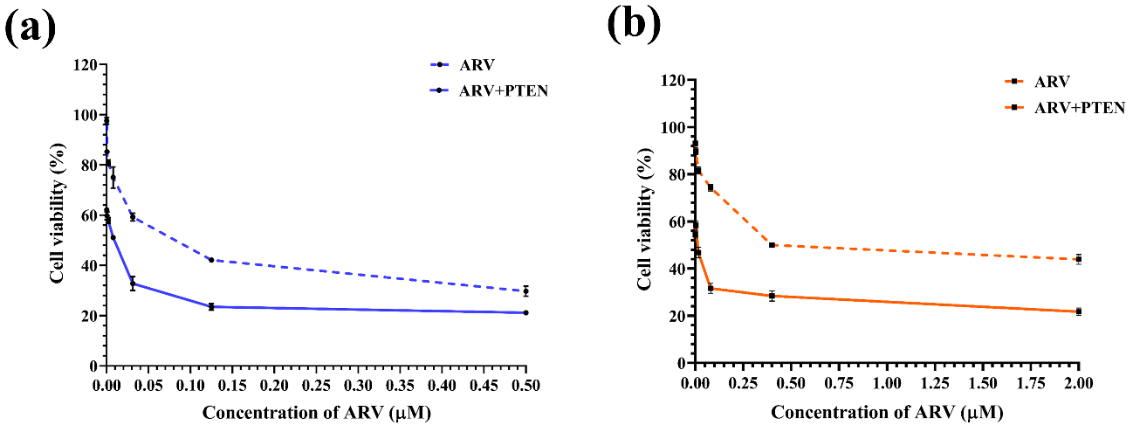

Fig. S5. Cytotoxicity curves of ARV and PTEN combination in VEM-resistant melanoma cell lines. *MTT cytotoxicity curves in* ***(a)*** *A375V (acquired) and* ***(b)*** *RPMI-7951 (intrinsic) resistant cell lines indicating strong synergism between ARV and PTEN in BRAFi-resistant melanoma. Control: Non-treated cells. (n = 6, Data are represented as mean ± SD).*

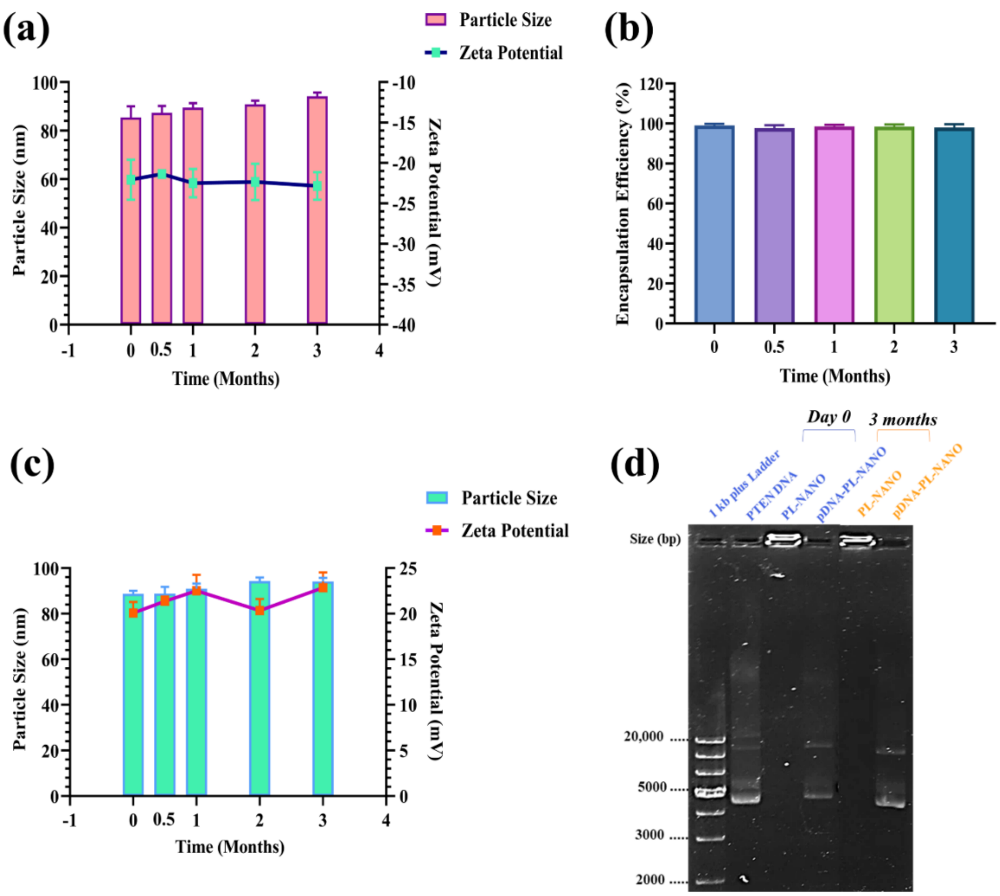

Fig. S6. Stability of AL-NANO and PL-NANO nanoformulations. (a) *Particle size and zeta potential of AL-NANO at different time points indicating no significant difference.* ***(b)*** *Entrapment efficiency of ARV in AL-NANO following 3 months at 4°C.* ***(c)*** *Particle size and zeta potential of PL-NANO depicting no substantial change at different time points.* ***(d)*** *Representative agarose gel electrophoresis image illustrating complete entrapment of PTEN plasmid in PL-NANO following 3 months at 4°C. (n = 3, Data are represented as mean ± SD).*
